# Updated assessment of risks and benefits of dolutegravir versus efavirenz in new antiretroviral treatment initiators in sub-Saharan Africa: modelling to inform treatment guidelines

**DOI:** 10.1016/S2352-3018(19)30400-X

**Published:** 2020-02-05

**Authors:** Andrew N Phillips, Loveleen Bansi-Matharu, Francois Venter, Diane Havlir, Anton Pozniak, Daniel R Kuritzkes, Annemarie Wensing, Jens D Lundgren, Deenan Pillay, John Mellors, Valentina Cambiano, Andreas Jahn, Tsitsi Apollo, Owen Mugurungi, David Ripin, Juliana Da Silva, Elliot Raizes, Nathan Ford, George K Siberry, Ravindra K Gupta, Ruanne Barnabas, Paul Revill, Jennifer Cohn, Alexandra Calmy, Silvia Bertagnolio

**Affiliations:** a Institute for Global Health, University College London, London, UK; b Ezintsha, Wits Reproductive Health and HIV Institute, University of the Witwatersrand, Johannesburg, South Africa; c School of Medicine, University of California San Francisco, San Francisco, CA, USA; d Chelsea and Westminster Hospital, London, UK; e London School of Hygiene & Tropical Medicine, London, UK; f Brigham and Women's Hospital, Harvard Medical School, Boston, MA, USA; g University Medical Center Utrecht, Utrecht, Netherlands; h Rigshospitalet, University of Copenhagen, Copenhagen, Denmark; i Africa Health Research Institute, Mtubatuba, South Africa; j Department of Medicine, Division of Infectious Diseases, University of Pittsburgh, Pittsburgh, PN, USA; k Ministry of Health, Lilongwe, Malawi; l Ministry of Health and Child Care, Harare, Zimbabwe; m Clinton Health Access Initiative, New York, NY, USA; n Centers for Disease Control and Prevention, Atlanta, GA, USA; o Centre for Infectious Disease Epidemiology and Research, University of Cape Town, Cape Town, South Africa; p WHO, Geneva, Switzerland; q Office of HIV/AIDS, Global Health Bureau, United States Agency for International Development, Arlington, VA, USA; r Cambridge Institute of Immunology and Infectious Diseases, University of Cambridge, Cambridge, UK; s Department of Global Health, University of Washington, Seattle, WA, USA; t Centre for Health Economics, University of York, York, UK; u Elizabeth Glaser Paediatric Health Foundation, Washington, DC, USA; v HIV/AIDS Unit, Geneva University Hospital, University of Geneva, Geneva, Switzerland

## Abstract

**Background:**

The integrase inhibitor dolutegravir is being considered in several countries in sub-Saharan Africa instead of efavirenz for people initiating antiretroviral therapy (ART) because of superior tolerability and a lower risk of resistance emergence. WHO requested updated modelling results for its 2019 Antiretroviral Guidelines update, which was restricted to the choice of dolutegravir or efavirenz in new ART initiators. In response to this request, we modelled the risks and benefits of alternative policies for initial first-line ART regimens.

**Methods:**

We updated an existing individual-based model of HIV transmission and progression in adults to consider information on the risk of neural tube defects in women taking dolutegravir at time of conception, as well as the effects of dolutegravir on weight gain. The model accounted for drug resistance in determining viral suppression, with consequences for clinical outcomes and mother-to-child transmission. We sampled distributions of parameters to create various epidemic setting scenarios, which reflected the diversity of epidemic and programmatic situations in sub-Saharan Africa. For each setting scenario, we considered the situation in 2018 and compared ART initiation policies of an efavirenz-based regimen in women intending pregnancy, and a dolutegravir-based regimen in others, and a dolutegravir-based regimen, including in women intending pregnancy. We considered predicted outcomes over a 20-year period from 2019 to 2039, used a 3% discount rate, and a cost-effectiveness threshold of US$500 per disability-adjusted life-year (DALY) averted.

**Findings:**

Considering updated information on risks and benefits, a policy of ART initiation with a dolutegravir-based regimen rather than an efavirenz-based regimen, including in women intending pregnancy, is predicted to bring population health benefits (10 990 DALYs averted per year) and to be cost-saving (by $2·9 million per year), leading to a reduction in the overall population burden of disease of 16 735 net DALYs per year for a country with an adult population size of 10 million. The policy involving ART initiation with a dolutegravir-based regimen in women intending pregnancy was cost-effective in 87% of our setting scenarios and this finding was robust in various sensitivity analyses, including around the potential negative effects of weight gain.

**Interpretation:**

In the context of a range of modelled setting scenarios in sub-Saharan Africa, we found that a policy of ART initiation with a dolutegravir-based regimen, including in women intending pregnancy, was predicted to bring population health benefits and be cost-effective, supporting WHO's strong recommendation for dolutegravir as a preferred drug for ART initiators.

**Funding:**

Bill & Melinda Gates Foundation.

## Introduction

The integrase inhibitor dolutegravir (within a fixed-dose combination of tenofovir disoproxil fumarate [tenofovir], lamivudine, and dolutegravir) is being considered for use (and, increasingly, used) in several countries in sub-Saharan Africa instead of efavirenz (in the form of tenofovir, lamivudine, and efavirenz) for people initiating antiretroviral therapy (ART) because of superior tolerability and a lower risk of resistance emergence.^1^, ^2^ However, there have been concerns over an increased risk of neural tube defects in babies conceived by women while taking dolutegravir.^3^ An approach to inform decision making is to quantify the risks and benefits of treatment policies through modelling to understand whether the benefits outweigh the risks. We previously reported such an analysis in the context of a policy of using dolutegravir in new ART initiators and switching from efavirenz to dolutegravir in people already on ART.^4^ However, this previous evaluation did not consider the specific question addressed in a 2019 WHO Antiretroviral Guidelines update,^5^ which was restricted to the choice of dolutegravir or efavirenz in new ART initiators. Furthermore, since our previous evaluation, additional data have emerged to inform risks and benefits.^2^, ^3^, ^6^ Updated data are available from the Tsepamo birth outcomes surveillance study in Botswana of neural tube defect risks with dolutegravir.^3^ Additionally, the NAMSAL^2^ and ADVANCE^6^ randomised trials have been fully published, which compared dolutegravir-based and efavirenz-based initial first-line ART in Africa.

**Research in context**

**Evidence before this study**

The integrase inhibitor dolutegravir has high potency and barrier to resistance, good tolerability, and low cost and has been adopted for use as a first-line antiretroviral therapy (ART) in some sub-Saharan African countries. In light of early data suggesting a possible risk of neural tube defects in babies of women taking dolutegravir at conception, in 2018, WHO made a conditional recommendation for dolutegravir as a preferred drug in ART initiators, with a note of caution when the drug was given to women of childbearing potential. Additionally, further data from randomised trials have emerged comparing dolutegravir-based first-line ART with efavirenz-based first line ART, including a new concern over weight gain with dolutegravir. Our team and another group previously modelled the risks and benefits of dolutegravir-based regimens. We searched Web of Science on May 15, 2019, and again on Nov 1, 2019, with no language restrictions, with the search terms “dolutegravir” AND “model*” and found no further published studies in any language that specifically modelled the risks and benefits of dolutegravir-based regimens for ART initiators.

**Added value of this study**

We modelled the balance of risks and benefits of ART initiation policies of an efavirenz-based regimen in women intending pregnancy and a dolutegravir-based regimen in others compared with a dolutegravir-based regimen in all ART starters, including in women intending pregnancy. We considered data published since our previous modelling of risks and benefits of dolutegravir-based regimens, including updated information on the possible small risk of neural tube defects in babies of women taking dolutegravir at conception and weight gain. We found that a policy of ART initiation with a dolutegravir-based regimen, including in women intending pregnancy, was predicted to bring net population health benefits and to be cost saving. Such a policy was cost-effective in 87% of our setting scenarios and this finding was robust in various sensitivity analyses.

**Implications of all the available evidence**

As shown by our analysis and a separate analysis by another modelling group, and in light of all evidence currently available, initiation of antiretroviral therapy with a dolutegravir-based regimen, including in women intending pregnancy, is predicted to bring population health benefits and be cost-effective. In July 2019, WHO updated its guidelines to make a strong recommendation for dolutegravir as a preferred drug in ART initiators for all HIV-positive adults. No recommendation currently exists for people who are already on a first-line efavirenz-based regimen as to whether a switch should be made to a dolutegravir-based regimen.

The ADVANCE trial^6^ gave rise to concern over substantial weight gain possibly caused by dolutegravir, particularly in women, for whom there was an average gain in weight of over 6 kg in 48 weeks in one dolutegravir group. In this study, we provide updated modelling results considering these updated data. These analyses were produced in response to a request from WHO to update modelling results for the 2019 Antiretroviral Guidelines revision.^5^

## Methods

### Modelling approach

We have previously described our model and how it was applied to consider risks and benefits of dolutegravir introduction.^4^ Briefly, the model is an individual-based model of sexual behaviour, transmission, and progression of HIV and the effect of ART in an adult population, with updates every 3 months. We sampled from distributions of parameters relating to, for example, rates of HIV testing, linkage and retention, ART adherence, resistance, interruption, extent of implementation of viral load monitoring, and rate of switching to second-line therapy after detected virological failure to create various epidemic setting scenarios, which reflect the diversity of epidemic and programmatic situations in sub-Saharan Africa (table 1). Values for several parameters relating to the potency of specific drugs, their toxicity profile, and the effects of toxicity on ART interruption, as well as drug resistance risk, the previous distribution of which reflects uncertainty over the values, were also varied across these setting scenarios. Descriptions and previous distributions of parameters are shown in the appendix (p 31).

**Table 1:** Setting scenarios in 2018 (n=1000)

	**Median (range)**^4^	Examples of observed data*
HIV prevalence (age 15–49 years)	13% (2·3–28·0)	Zimbabwe 2016 13%; Tanzania 2017 5%; Uganda 2017 6%; Lesotho 2017 24%; eSwatini 2017 27%; Malawi 2016 10%; Namibia 2017 12%; Zambia 2016 11%; Cameroon 2017 3·4%; Côte d'Ivoire 2017–18 2·5%
HIV incidence (age 15–49 years per 100 person-years)	0·90 (0·10–2·86)	Malawi 2016 0·37; Zambia 2016 0·66; Zimbabwe 2016 0·45; Lesotho 2017 1·55; Namibia 2016 0·40; Swaziland 2017 1·48, Tanzania 2017 0·27; Cameroon 2017 0·27
Proportion of HIV-positive people diagnosed	84% (50–97); 76% men, 90% women)	Malawi 2016 77%; Zambia 2016 67%; Zimbabwe 2016 74%; Namibia 2017 86%; Tanzania 2017 52%; Ethiopia 2018 72%; Côte d'Ivoire 2017–18 37%; Cameroon 2017 47%
Proportion of diagnosed HIV-positive people on ART	88% (68–99)	Lesotho 2016–17 92%; South Africa 2017 71%; eSwatini 2016–17 87%; Namibia 2017 96%; Zambia 2016 87%; Tanzania 2016–17 94%; Ethiopia 99%; Malawi 2016 91%; Uganda 2016–17 90%; Cameroon 2017 91%; Zimbabwe 2016 87%; Côte d'Ivoire 2017–18 88%; Cameroon 2017 91%
Proportion of all HIV-positive people with viral load <1000 copies per mL	60% (36–82)	Zambia 2016 60%; Malawi 2016 68%; Zimbabwe 2016 60%; Swaziland 2017 73%; Lesotho 2017 68%; Tanzania 2017 52%; Uganda 2017 60%; Namibia 2017 77%; Ethiopia 2018 70%; Côte d'Ivoire 2017–18 40%; Cameroon 2017 47%
Proportion of ART-experienced people who have started second-line ART	4% (0·6–31·0)	Malawi ∼3% (Malawi Ministry of Health Quarterly Reports)
Proportion of people on ART with viral load <1000 copies per mL	88% (72–97); 85% men, 89% women	Zambia 2016 men 88%, women 90%; Malawi 2016 men 90%, women 92%; Zimbabwe 2016 men 84%, women 88%; Namibia 2017 men 92%, women 90%; Tanzania 2017 men 89%, women 83%; Ethiopia 2018 men 95%, women 87%; Côte d'Ivoire 2017–18 76%; Cameroon 2017 80%
Proportion of people on ART with CD4 <500 cells per μL (in 2016)	44% (30–70)	eSwatini 40% 2016–17; Malawi 52% 2016; Tanzania 2017–18 55%; Zambia 2016 59%
Proportions of ART-naive ART initiators with non-nucleoside reverse transcriptase inhibitor resistance	8% (1–30)	Angola 2012 14%; Botswana 2016 8%; South Africa 2017 14%; Zimbabwe 2015 10%; Namibia 9%; Uganda 2016 16%; Cameroon 8% (HIV drug resistance report 2017^7^)
Mother-to-child transmission rate	9% (3–25)	(including breastfeeding period) Botswana 5%, South Africa 5%; Namibia 6%; Uganda 8%; Zimbabwe 7%; Malawi 9%; Tanzania 12%; Ethiopia 21%; Côte d'Ivoire 16%; Cameroon 15% (UNAIDS 2019^8^)
Proportion of women aged 15–65 years giving birth per year	12% (5–19)	South Africa 6%; Botswana 7%; Namibia 10%; Malawi 15%; Zimbabwe 12%; Uganda 18%; Tanzania 16%; Ethiopia 12%; Côte d'Ivoire 15%, Cameroon 14% (2019 Revision of World Population Prospects^9^)

ART=antiretroviral therapy.

* All data from Population Health Impact Assessment surveys, Human Sciences Research Council South Africa National HIV survey,^10^ or Botswana Combination Prevention Project (2013–15),^11^ unless otherwise stated.

### Modelling of drug activity and resistance

The ongoing effect of an ART regimen in a modelled person depends on current adherence and the current level of activity of the overall regimen, dependent on the potency (activity against HIV when resistance is not present) of each drug and whether resistance mutations relevant to that drug are present (appendix p 5). Based on the results from the ADVANCE^6^ and NAMSAL^2^ trials, we assumed that dolutegravir and efavirenz have equal potency (1·5; ie, when no resistance mutations are present they contribute 1·5 active drugs to the overall drug activity; all nucleoside analogue reverse-transcriptase inhibitors have potency 1). Drug resistance has a low chance of arising in any regimen with three or more active drugs when adherence is high, but the risk becomes more substantial with lower current regimen activity and with low-to-moderate adherence. We maintained our previous assumption that the rate of resistance to dolutegravir (for a given level of regimen activity and adherence) is 13 times lower than efavirenz, with a small proportion of setting scenarios with a four times lower risk only.^4^ Although the relative rate of resistance between drugs was unchanged from our previous work, given the high levels of viral suppression in the ADVANCE trial,^6^ we considered that the absolute rate of resistance to all drugs could be lower than we originally assumed and thus included a higher proportion of setting scenarios with half the base rate of resistance (50% rate of resistance compared with base case in 54% of setting scenarios). Our base assumption was that mutations Gly190Ala and Tyr181Cys reduce efavirenz activity by 75% and Lys103Asn reduces efavirenz activity by 100%. The above assumption holds for 76% of setting scenarios—for 20% of setting scenarios activity level is reduced by 50% if any of the mutations are present and for 4% of setting scenarios activity level is reduced by 75%. With these assumptions, the mean odds ratio (OR) for viral load greater than 1000 copies per mL at 1 year from start of ART associated with pre-treatment non-nucleoside reverse transcriptase inhibitor drug resistance is 3·3 (1·75 in a sensitivity analysis).

### Modelling of mother-to-child transmission and neural tube defects

Our assumption of risk of mother-to-child transmission was unchanged from our previous analysis.^4^ We assumed that for each subsequent year of the time horizon, 0·1 disability-adjusted life-years (DALYs) are experienced for an HIV-positive baby and a cost of US$160 incurred as a conservative estimate of ART care costs, unchanged from our previous publication.^4^ We updated the risk of neural tube defects in pregnant women due to dolutegravir to 0·22% (0·30% minus the 0·08% background rate, compared with 0·58% previously).^3^, ^4^ As before, for a woman having a baby with a neural tube defect an extra DALY was assumed to be incurred for each subsequent year of the time horizon due to loss of years of the child's life.^4^ No additional costs were assumed. The presence of current toxicity increased the risk of drug interruption and increased weight was considered an additional toxic effect of dolutegravir.

### Modelling potential effects of weight gain due to dolutegravir

We included effects on morbidity and mortality because of the effects of weight on conditions such as diabetes, as well as possible effects of potential increases in bodyweight on babies of pregnant women. For mortality, we based our assumptions on data concerning the observed relationship between body-mass index (BMI) and all-cause mortality, both in HIV-positive people and the general population,^12^, ^13^ as suggested could be possible, based on a pooled analysis of BMI and cardiometabolic multimorbidity.^14^ We assumed that a proportion (50% or 75%^15^, ^16^) of people have a BMI greater than 23 kg/m^2^ at dolutegravir initiation, such that a rise in the order of that seen in the ADVANCE trial^6^ for people on tenofovir, lamivudine, and dolutegravir might be clinically significant.^3^ We sampled a range of values for the excess risk of non-HIV mortality from 1·00 times to 1·25 times (appendix p 36). Additionally, many non-AIDS deaths will be associated with several years of previous morbidity, which reduces health (incurs DALYs) and incurs costs to the health-care system. We accounted for these by assuming a one DALY increment for a person who dies from a non-AIDS cause (not withstanding discounting), reflecting 10 years of living with a disability weight of 0·1 (eg, the same as the weight for diabetic foot; moderate angina or moderate heart failure has a disability weight of 0·07^17^). Regarding the cost of management of these conditions, we have a one-off mean cost of the years of health care for morbidity for all non-AIDS deaths. We assumed a value of $1000, informed by health-care budgets for non-communicable diseases in sub-Saharan Africa.^18^, ^19^ There is also a potential excess of neonatal death caused by dolutegravir-induced weight gain. The overall risk of neonatal death for women of normal bodyweight is around 1·6%.^20^ The rate ratio for neonatal death per unit of higher BMI for women with BMI above 23 kg/m^2^ is around 1·05, so the absolute excess risk due to a 2·5 kg increase in weight is 0·08% ([1·05 – 1] × 1·6%). As there is also a potential excess risk of stillbirth, for which the absolute risk is similar, the overall risk of stillbirth or neonatal death is around 0·16%. We sampled the risk of stillbirth or neonatal death from 0·05% to 0·30%.

### Policies compared, DALYs, and net DALYs

For each setting scenario, we considered the situation in 2018 and compared ART initiation policies of initiating ART with tenofovir, lamivudine, and efavirenz in women intending pregnancy, and initiating ART with tenofovir, lamivudine, and dolutegravir in others and initiating ART with tenofovir, lamivudine, and dolutegravir, including in women intending pregnancy. We considered predicted outcomes over a 20-year period from 2019 to 2039. We calculated DALYs for the entire adult population, assuming that a woman having a child with a neural tube defect incurs an extra DALY per year for the remainder of the time horizon and also accounting for mother-to-child transmission. Unless stated otherwise, absolute numbers of health-related events, costs, and DALYs are relevant for a country of population size of around 10 million adults in 2018. As in our previous study,^4^ cost-effectiveness analyses were done from a health-care perspective, costs and health outcomes were both discounted to present US$ values at 3% per annum, and a cost-effectiveness threshold of $500 per DALY averted was used. We used this threshold to calculate net DALYs averted (net DALYs averted=DALYs averted + [difference in costs ÷ cost-effectiveness threshold]). Net DALYs accounted for the health consequences of the difference in costs as well as the difference in health and reflected the effect of a policy on the overall population burden of disease. Costs and disability weights are shown in the appendix (p 38).

### Role of the funding source

The funder of the study had no role in study design, modelling approach, data interpretation, or writing of the report. The corresponding author had full access to all model programs and outputs in the study and had final responsibility for the decision to submit for publication.

## Results

Our model predicted outcomes over 20 years for the two policies (Table 2, Table 3). The mean proportion of women with viral suppression 1 year after the start of ART was around 6% higher for the policy of ART initiation with tenofovir, lamivudine, and dolutegravir in women intending pregnancy compared with the policy of ART initiation with tenofovir, lamivudine, and efavirenz in women intending pregnancy, with a 5% difference in the overall proportion of women on ART with viral suppression (table 2). There was also a slightly higher CD4 count rise 1 year from the start of ART with the policy of tenofovir, lamivudine, and dolutegravir in women intending pregnancy (table 2). These differences led to a lower HIV-related death rate for the policy of tenofovir, lamivudine, and dolutegravir in women intending pregnancy (table 2). The non-HIV-related death rate was slightly higher for the policy of tenofovir, lamivudine, and dolutegravir in women intending pregnancy because of the effect of weight gain (table 2). Considering outcomes for babies, the higher number of neural tube defects and stillbirths and neonatal deaths associated with higher weight in mothers was substantially outweighed by the lower number of children born with HIV with the policy of tenofovir, lamivudine, and dolutegravir in women intending pregnancy (table 2).

**Table 2:** Predicted effects of policy options over 20 years

		ART initiation with tenofovir, lamivudine, and efavirenz in women intending pregnancy*	ART initiation with tenofovir, lamivudine, and dolutegravir in women intending pregnancy*
Proportion of women intending pregnancy on ART who are on†			
	Dolutegravir	0%	48%, 48% (48 to 48; 37 to 57)
	Efavirenz	92%	46%, −46% (−46 to −46; −56 to −33)
	Atazanavir‡	8%	6%, −2% (−2 to −2; −7 to 0)
	Tenofovir	92%	94%, 2% (2 to 2; 0 to 7)
	Lamivudine	100%	100%, 0% (0 to 0; 0 to 0)
	Zidovudine	8%	6%, −2% (−2 to −2; −7 to 0)
Proportion of women intending pregnancy with viral load <1000 copies per mL 12 months from ART initiation†		74%	80%, 6% (6 to 6; 0 to 12)
Proportion of women intending pregnancy on ART with viral load <1000 copies per mL 12 months from ART initiation†		82%	88%, 6% (6 to 6; 1 to 13)
Proportion of women intending pregnancy on ART with viral load <1000 copies per mL†		84%	89%, 5% (5 to 5; 1 to 10)
In women intending pregnancy on ART at 12 months from start of ART, change in CD4 cell count per μL†		132	151, 20 (19 to 20; 1 to 43)
Proportion of all HIV-positive people with:†			
	Efavirenz resistance	23%	19%, −4% (−4 to −4; −9 to −1)
	Dolutegravir resistance	1%	2%, 1% (1 to 1; 0 to 3)
Death rate in women intending pregnancy on ART (per 100 person-years)†		1·78	1·42, −0·36 (−0·38 to −0·034; −1·00 to −0·01)
	HIV-related	1·03	0·65, −0·38 (−0·40 to −0·036; −1·02 to −0·05)
	Non-HIV-related	0·76	0·78, 0·02 (0·02 to 0·02; 0·07 to 0·12)
Outcomes for babies—annual number of cases of:†			
	Neural tube defects	1	73, 72 (69 to 74; 15 to 156)
	Mother-to-child transmission	9650	8150, −1500 (−1400 to −1600; −4450 to 0)
	Excess stillbirths or neonatal death due to dolutegravir-induced increase in BMI	1	43, 42 (40 to 45; 0 to 124)
Mean total DALYs averted per year compared with tenofovir, lamivudine, and efavirenz (DALYs in adults, DALYs due to neural tube defects and other dolutegravir-induced obesity-related adverse neonatal events)		..	10 990 (10 248, 11 732; −7075 to 31 840)
Breakdown of mean total DALYs averted per year compared with tenofovir, lamivudine, and efavirenz			
	Before accounting for neural tube defects, prevention of mother-to-child transmission, and morbidity effects of weight gain	..	10 970
	Neural tube defects	..	−270
	Mother-to-child transmission	..	510
	Excess risk of stillbirth or neonatal death due to dolutegravir-induced increase in BMI	..	−170
	Pre-death morbidity due to weight gain	..	−40
Mean cost over 3-month periods from 2019 to 2039 (millions of US$, discounted at 3% per year)§			
	Total	195·7	192·9 (difference −2·9)
	HIV tests	10·8	10·7
	Efavirenz	18·2	13·0
	Dolutegravir	4·7	10·2
	Lamivudine	19·8	19·9
	Tenofovir	27·4	27·9
	Zidovudine	4·4	3·9
	Atazanavir	13·1	11·4
	Clinic visits (non-ART programme costs)	55·0	55·1
	Treatment or care for WHO stage 3 or 4 events	12·4	11·5
	Viral load tests	7·4	7·3
	Treatment for children because of mother-to-child transmission	6·9	6·3
	Non-AIDS pre-death morbidity	14·4	14·5
	CD4 counts	0·5	0·5
	Adherence counselling	0·2	0·1
	Cost in staff time of switching to second-line ART	0·1	0·1
Net DALYs averted per year		..	16 735

ART=antiretroviral therapy. BMI=body-mass index. DALY=disability-adjusted life-year.

* ART initiated in men and women not wanting (more) children is tenofovir, lamivudine, and dolutegravir.

† Data in the first column are the mean over 3-month periods from 2019 to 2039 for each policy. Data in the second column are the mean, mean absolute difference (95% CI; 90% range reflecting variation across setting scenarios) compared with the policy of first-line tenofovir, lamivudine, and efavirenz in women intending pregnancy.

‡ We assume atazanavir is used as the protease inhibitor in second-line ART.

§ Cost breakdown by regimen policy (for all adults in context of a population of 10 million adults). Tenofovir, lamivudine, and dolutegravir and tenofovir, lamivudine, and efavirenz are assumed to cost $75 per year, atazanavir $265 per year (Global Fund procurement list of drug costs, July 2018^21^). Viral load test costs reflect that in some setting scenarios viral load testing is minimally implemented—the total cost is double this mean cost if fully implemented.

**Table 3:** Proportion of setting scenarios in which tenofovir, lamivudine, and dolutegravir brings direct health benefit or is cost-effective, over 20 years

		**Tenofovir, lamivudine, and dolutegravir brings direct health benefit (ie, DALYs averted)**	**Tenofovir, lamivudine, and dolutegravir is cost-effective (ie, net DALYs are averted)**
Overall		83%	87%
Sensitivity analyses—restricted to setting scenarios for which:			
	Proportion of ART-naive ART initiators with non-nucleoside reverse transcriptase inhibitor resistance in 2018 is in the range 10–30%	89%	94%
	Proportion of ART-naive ART initiators with non-nucleoside reverse transcriptase inhibitor resistance in 2018 <5%	79%	82%
	HIV prevalence 10–28% in 2018	86%	91%
	Proportion of people on ART with viral load <1000 copies per mL 90–97% in 2018	71%	77**%**
	Proportion of people on ART with viral load <1000 copies per mL 72–85% in 2018	93%	95%
	Viral load monitoring and switching to second-line ART operating well*	75%	87%
	Viral load monitoring and switching to second-line ART operating poorly or not at all†	89%	87%
	16–19% of women giving birth per year	78%	82%
	5–12% of women giving birth per year	85%	89%
	1·25 times increased risk of non-HIV death due to weight gain	76%	85%
	≤1·05 times increased risk of non-HIV death due to weight gain	85%	88%
	Absolute additional risk of stillbirth or neonatal death due to weight gain is 0·05%	82%	85%
	Absolute additional risk of stillbirth or neonatal death due to weight gain is 0·30%	82%	84%
	Risk of neural tube defects due to dolutegravir is 0·61%	82%	87%
	Reduced impact of non-nucleoside reverse transcriptase inhibitor mutations on efavirenz activity‡	73%	78%
	Halving of the risk of resistance mutations emerging for all drugs	82%	86%

ART=antiretroviral therapy. DALY=disability-adjusted life-year.

* Viral load monitoring and switching to second-line ART operating poorly means the probability of each scheduled viral load test being done is 0·1 or 0; the probability per 3 months of a switch to second-line ART once failure criteria are met is 0 or 0·2.

† Viral load monitoring and switching to second-line ART operating well means the probability of each scheduled viral load test being done is 0·85; the probability per 3 months of a switch to second-line ART once failure criteria are met is 0·5.

‡ With this assumption, the mean odds ratio for viral load >1000 copies per mL at 1 year from the start of ART associated with pre-treatment on nucleoside reverse transcriptase inhibitor resistance is 1·75, compared with 3·3 for the overall result (appendix p 36).

Considering the whole adult population, more DALYs are averted with the policy of ART initiation with tenofovir, lamivudine, and dolutegravir in women intending pregnancy compared with the policy of tenofovir, lamivudine, and efavirenz. Overall costs were slightly lower with the policy of ART initiation with tenofovir, lamivudine, and dolutegravir in women intending pregnancy compared with the policy of ART initiation with tenofovir, lamivudine, and efavirenz in women intending pregnancy, driven mainly by lower use of the second-line drugs atazanavir and zidovudine and lower costs of care for HIV-related conditions (table 2). Net DALYs averted per year show a similar pattern to DALYs averted but since costs are averted with the policy of tenofovir, lamivudine, and dolutegravir in women intending pregnancy, the benefits are greater (table 2).

Overall, the policy of ART initiation with tenofovir, lamivudine, and dolutegravir in women intending pregnancy produced more health than the policy of ART initiation with tenofovir, lamivudine, and efavirenz in women intending pregnancy (DALYs were averted) in 83% of setting scenarios and was cost-effective (net DALYs averted) in 87% of setting scenarios (table 3). In sensitivity analyses restricted to setting scenarios with particular characteristics, the policy of tenofovir, lamivudine, and dolutegravir in women intending pregnancy remained the most cost-effective in over 75% of setting scenarios (table 3). A corresponding multivariable model is shown in the appendix (p 2). We also did a sensitivity analysis to assess policies over a 50-year time horizon instead of the 20-year time horizon in our primary analyses. The proportion of setting scenarios in which tenofovir, lamivudine, and dolutegravir brought direct health benefit was 94% and the proportion in which it was cost-effective was 98%.

## Discussion

The results of this study add to our previous evaluation of the risks and benefits of adoption of dolutegravir within antiretroviral regimens.^4^ Considering data from the ADVANCE^6^ and NAMSAL^2^ trials and the Tsepamo study,^3^ we found that the policy of ART initiation with tenofovir, lamivudine, and dolutegravir in women intending pregnancy is predicted to lead to more health life years compared with the policy of ART initiation with tenofovir, lamivudine, and efavirenz in women intending pregnancy and is cost-effective. Because of the results of the ADVANCE^6^ and NAMSAL^2^ trials, we modified our previous assumptions such that the potency of efavirenz and dolutegravir was taken as equal, when we previously considered that potency was likely to be higher for dolutegravir. We also accounted for a possible effect of weight gain due to dolutegravir on non-AIDS morbidity and mortality. Nevertheless, the policy of ART initiation with tenofovir, lamivudine, and dolutegravir in women intending pregnancy was more cost-effective in 87% of our setting scenarios and this result was robust across several sensitivity analyses in which our assumptions were varied within plausible bounds. The predicted health benefits of tenofovir, lamivudine, and dolutegravir are substantial—eg, the decreased death rate in women on ART of 0·36 per 100 person-years could translate into over 1800 deaths in women per year for Zimbabwe. Our results are consistent with those of a modelling analysis from the CEPAC study.^22^ These results were presented for consideration by the WHO guidelines development group, which reviewed all the available evidence and made a strong recommendation for dolutegravir as a preferred drug in ART initiators.^5^

Studies have consistently shown that dolutegravir leads to lower rates of discontinuation than efavirenz.^1^, ^2^, ^6^ In our analysis, the predicted health benefits of the policy of ART initiation with tenofovir, lamivudine, and dolutegravir in women intending pregnancy are mainly driven by the higher genetic barrier to resistance of dolutegravir compared with efavirenz, rather than this tolerability benefit. It is difficult to ascertain how the tolerability benefits of dolutegravir play out in the context of settings in which drug switching is not readily available because of interrupted procurement of efavirenz, and where the choice might be between continuing the current regimen or stopping ART altogether. Additionally, the likelihood of subsequent reinitiation of ART at some point after interruption should be considered, and we assume that, in a person who has interrupted ART, the subsequent development of an HIV-related condition (WHO stage 3 or stage 4) leads to an increased probability that they will resume therapy. If rates of stopping all ART are substantially greater for people on tenofovir, lamivudine, and efavirenz than on tenofovir, lamivudine, and dolutegravir, the benefits of tenofovir, lamivudine, and dolutegravir are likely to be greater than those we describe in this study. Side-effects of drugs, including weight gain, and their effect on ART use patterns should continue to be monitored carefully over the long term.

Our assumptions about the potential effects of weight gain on non-AIDS mortality include effects (1·25 times for the duration of time on dolutegravir) beyond what would be predicted on the basis of observed associations between BMI and mortality.^12^, ^13^ We took a conservative approach because of uncertainty over consequence of dolutegravir-induced weight gain. We also considered that weight gain could lead to an increased risk of stillbirth or neonatal mortality in the babies of mothers on the drug, which might be particularly relevant given the larger weight gain observed in women.^6^ However, we emphasise that it is unclear whether any such effect exists and we included this effect to consider a worst case scenario for dolutegravir.

Modelling studies inevitably have the limitation that they are based on model design and assumptions, but our overall conclusion seems robust to alternative assumptions within plausible limits. Although our analysis has been widely considered and commented on, we cannot definitively rule out that there are other alternative assumptions that we did not consider. Additionally, monitoring of new data on outcomes from use of dolutegravir as these emerge will be important, including on weight gain, resistance emergence, and neural tube defects, and updates to analyses should be made if required. Our evaluation pertains to people starting ART and does not address the question of risks and benefits of switching from tenofovir, lamivudine, and efavirenz to tenofovir, lamivudine, and dolutegravir in people already on ART.

In conclusion, in the context of a range of modelled setting scenarios in sub-Saharan Africa, we found that initiation of ART with a dolutegravir-based regimen was predicted to bring population health benefits and be cost-effective, supporting WHO's strong recommendation as a preferred drug in ART initiators, including women intending pregnancy.

## Data sharing

Model programs are accessible in Figshare.
