# Supplementary material for: Updated assessment of risks and benefits of dolutegravir versus efavirenz in new antiretroviral treatment initiators in sub-Saharan Africa: modelling to inform treatment guidelines
Source: Lancet HIV. 2020 Feb 5;7(3):e193–200. doi: 10.1016/S2352-3018(19)30400-X (PMC7167509; doi:10.1016/S2352-3018(19)30400-X)
Supplement: Supplementary appendix [file mmc1.pdf]

THE LANCET
HIV

Supplementary appendix

This appendix formed part of the original submission and has been peer reviewed. We post it as supplied by the authors.

Supplement to: Phillips AN, Bansi-Matharu L, Venter F, et al. Updated assessment of risks and benefits of dolutegravir versus efavirenz in new antiretroviral treatment initiators in sub-Saharan Africa: modelling to inform treatment guidelines. *Lancet HIV* 2020; published online Feb 5. https://doi.org/10.1016/S2352-3018(19)30400-X.

Appendix

Updated assessment of Risks and benefits of dolutegravir versus efavirenz in new antiretroviral treatment initiators in sub-Saharan Africa: modelling to inform treatment guidelines

1. Additional Results Tables Page 2
2. Model Details Page 4

Additional Results Table 1. Associations between key characteristics and cost effectiveness of TLD based on analysis of 1000 setting scenarios (n=873 where TLD is cost effective).

		Odds ratio	95% CI
**Of ART-naïve ART initiators % with NNRTI resistance in 2018**	< 5%	1.00	----
	5% - 9.9%	1.11	0.69 - 1.79
	**≥ 10%**	**1.98**	**1.11 - 3.54**
**HIV prevalence in 2018**	< 10%	1.00	----
	**≥ 10%**	**3.58**	**2.36 – 5.43**
**Of people on ART, proportion with VL < 1000 in 2018**	< 72%	1.00	----
	72% - 89.9%	0.62	0.33 – 1.17
	**≥ 90%**	**0.22**	**0.12 – 0.39**
Viral load monitoring and switching to 2nd line ART operating performance level	high	1.00	----
	medium	0.73	0.38 – 1.41
	low	0.68	0.33 – 1.39
**% of women giving birth per year**	< 12%	1.00	----
	12% - 15.9%	0.77	0.49 – 1.22
	**≥ 16%**	**0.52**	**0.29 – 0.91**
Fold-increased risk of non-HIV death due to weight gain	≤ 1.05	1.00	----
	1.07 – 1.15	0.95	0.60 – 1.51
	1.25	0.91	0.50 – 1.65
Absolute additional risk of stillbirth/neonatal death weight gain	0.005%	1.00	----
	0.015% - 0.02%	1.43	0.86 – 2.39
	0.03%	0.90	0.50 – 1.62
**Reduced impact of NNRTI mutations on efavirenz activity +**	No	1.00	**----**
	**Yes**	**0.40**	**0.26 – 0.60**
Halving of the risk of resistance mutations emerging for all drugs	Base assumption	1.00	----
	0.5 fold lower rate	0.95	0.63 – 1.43

+ With these assumptions the average odds ratio for VL > 1000 copies/mL at 1 year from start of ART associated with pre-treatment NNRTI drug resistance is 1.75 compared with 3.3 for the overall result. (See Appendix Table S10 for details)

Additional Results Table 2. Associations between key characteristics and cost effectiveness of TLD based on a data set of 2000 setting scenarios, consisting of the data set of 1000 setting scenarios duplicated, with 1000 based on the base NTD risk, and 1000 based on higher NTD risk (upper 95% confidence limit). Thus in this Table, unlike the one above, we include NTD risk as a potential predictor.

		Odds ratio
Of ART-naïve ART initiators % with NNRTI resistance in 2018	< 5%<br>5% - 9.9%<br>≥ 10%	1.00<br>1.13<br>1.89
HIV prevalence in 2018	< 10%<br>≥ 10%	1.00<br>3.73
Of people on ART, proportion with VL < 1000 in 2018	< 72%<br>72% - 89.9%<br>≥ 90%	1.00<br>0.57<br>0.21
Viral load monitoring and switching to 2nd line ART operating performance level	high<br>medium<br>low	1.00<br>0.72<br>0.62
% of women giving birth per year	< 12%<br>12% - 15.9%<br>≥ 16%	1.00<br>0.80<br>0.53
Fold-increased risk of non-HIV death due to weight gain	≤ 1.05<br>1.07 – 1.15<br>1.25	1.00<br>0.95<br>0.92
Absolute additional risk of stillbirth/neonatal death weight gain	0.005%<br>0.015% - 0.02%<br>0.03%	1.00<br>1.36<br>0.86
Reduced impact of NNRTI mutations on efavirenz activity +	No<br>Yes	1.00<br>0.40
Halving of the risk of resistance mutations emerging for all drugs	Base assumption<br>0.5 fold lower rate	1.00<br>0.99
NTD risk	0.22%<br>0.61%	1.00<br>0.93

+ With these assumptions the average odds ratio for VL > 1000 copies/mL at 1 year from start of ART associated with pre-treatment NNRTI drug resistance is 1.75 compared with 3.3 for the overall result. (See Appendix Table S10 for details)

Model details

Here we describe details of the modelling in relation to drug resistance and the effect of ART as well as pregnancy. Details of modelling of demographics, sexual behaviour, HIV transmission and HIV testing are explained in supplement to a recent paper[1] and can be found here: https://www.thelancet.com/cms/10.1016/S2352-3018(17)30190-X/attachment/02742987-df48-4372-8e4a-43888c2ec1e8/mmc1.pdf Before giving full details we show (Figure S1) outputs of the model relating to outcomes by 1, 3 and 10 years from initiation of first line ART with either an efavirenz, atazanavir or dolutegravir based regimen (each with tenofovir and 3TC) in the absence of any switching in drug regimen. This illustrates the combined effects of the model assumptions which are described below. This is in the context of adherence profile B (see below for different adherence profiles considered), and it is for a situation with which there is no pre-ART NNRTI resistance.

Figure S1. Illustration of assumptions on effectiveness of efaviren and dolutegravir-containing 1st line regimens. Outcomes at 1, 3 and 10 years in absence of any switching to second line

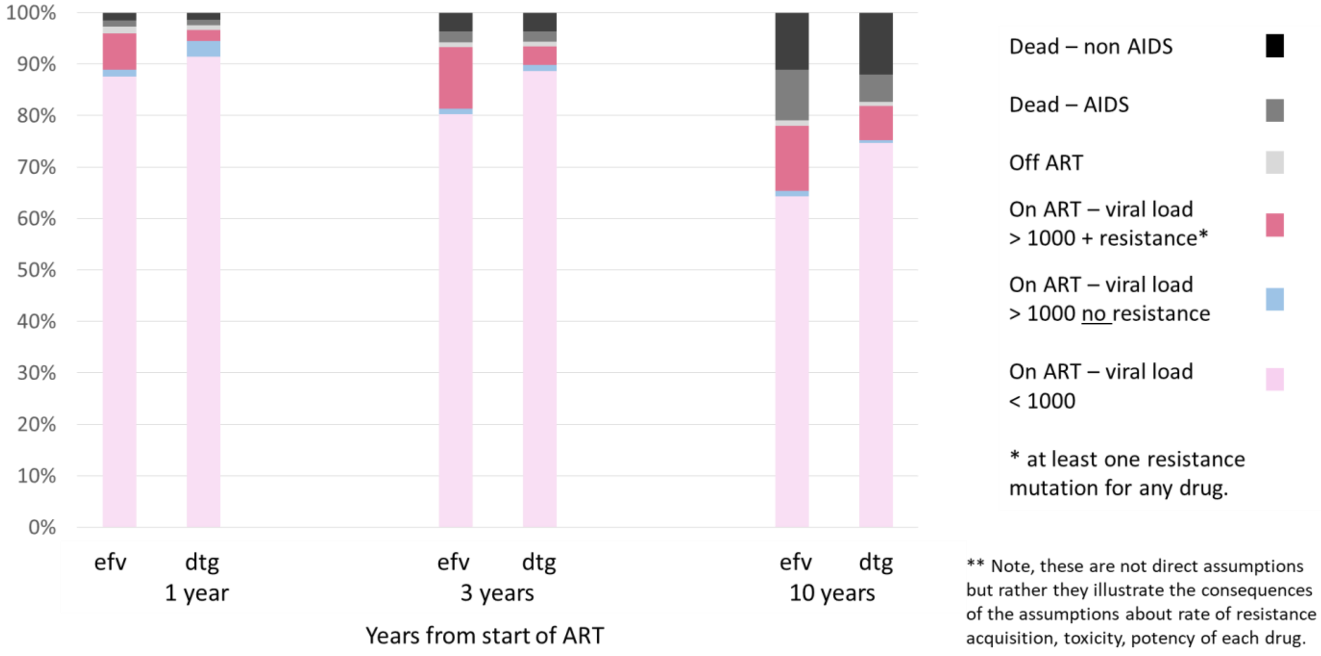

Throughout the sections below we introduce parameters which are indicated in italics. For those parameters for which a value is sampled the distribution is indicated at the end of this document.

Modelling the effect of ART

The structure of how the relationship between ART adherence, viral load, development of resistance, CD4 count and risk of death is modelled is illustrated in Figure S2 below. The adherence level - the determination of which is described in detail below - influences the risk of acquisition of new mutations as well as having a direct effect on the viral load and CD4 count. Acquisition of resistance mutations impacts on the total activity level of the regimen, calculated as the sum of the activity level of the drugs, akin to what is sometimes referred as a "genotypic sensitivity score". This, in turn, is a further determinant of the risk of new mutations arising. Distinction is made for each resistance mutation as to whether it is only present in minority virus (which can occur if the patient has a mutation present but is not taking a drug that selects for that mutation), so the

mutation is assumed not transmissible, or if it is present in majority virus. Failure of the current line of ART is determined by CD4 count or viral load or clinical disease, depending on the monitoring strategy being implemented (in the current paper we assumed viral load monitoring from 2016, but with various levels of implementation), and this triggers a switch to the next line of ART (if assumed available, and often with a delay). The following sections provide further details, including how adherence levels are determined and how they influence the viral load, risk of resistance and the CD4 count. We also explain the modelling of ART interruption and loss to follow-up. We provide references to papers that have been used to inform the approach. It should be noted though that parameter values used in the model are rarely extracted directly from any one paper, they are values that are arrived at based on their ability to generally reproduce outputs that are consistent with observed estimates, as illustrated below.

Figure S2. Overview of the modelling of the effect of ART, highlighting the role of adherence.

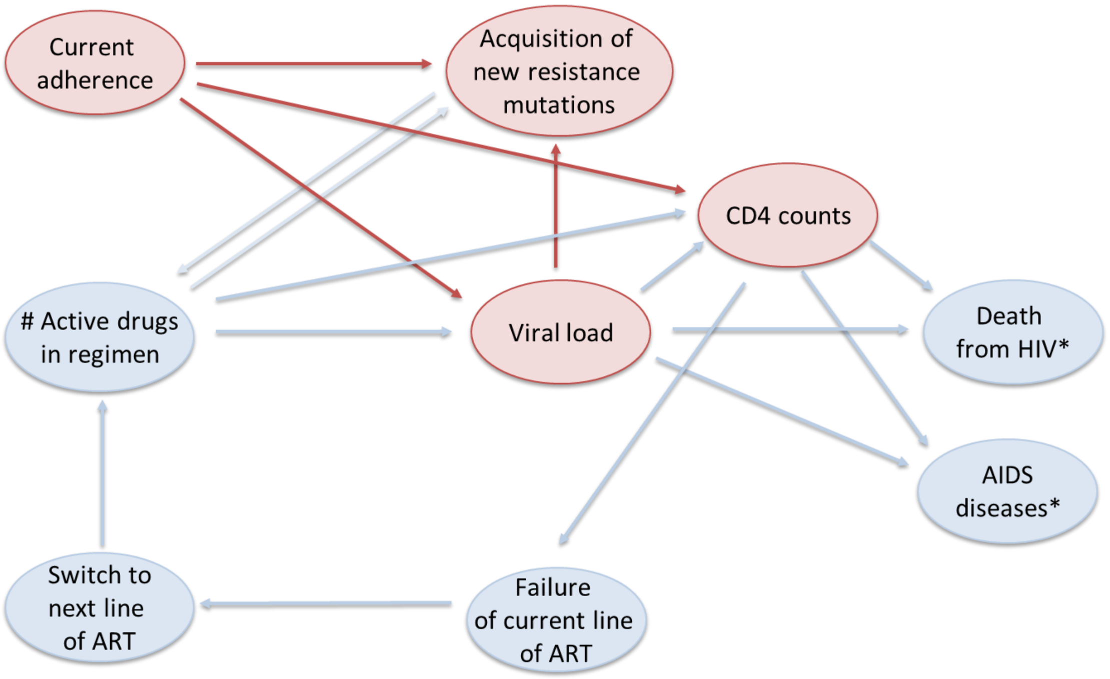

*influenced by age and PCP prophylaxis also

Initiation of ART

It is assumed ART became available in 2004. Eligibility for ART initiation in people diagnosed with HIV before 2003 is determined by the development of a WHO 4 or TB event. From 2004 to 2010, eligibility for ART initiation is determined by a measured CD4 count < 200 (in the last year) or the development of a WHO 4 event or TB and from 2011 to 2014 by a CD4 count <350 or a WHO 4 event. From 2011 onwards, pregnancy (option B+) is also an indicator for ART initiation. From 2014, ART initiation was indicated also based on a CD4 count < 500. From 2017 onwards, all people diagnosed with HIV are eligible for treatment. For people that are eligible to be initiated on treatment the probability that ART initiation occurs is determined by sampling from a Uniform (0,1) distribution and determining whether this is below the value for *pr_art_init*.

Switch to second line after failure of first line ART

The probability of switching per 3 month period after the criterion for failure of first line ART is met is *pr_switch_line*. The switch rate is likely to vary substantially by setting[2,3]

Adherence pattern

The model specifies a current adherence level (i.e. for the current 3 month period) for people on ART, a value between 0%-100%. We first give a brief description of the approach and then give further detail. Since the model updates in 3 month periods, short term interruptions of days or a few weeks are treated as sub-optimal average adherence during the 3 month period. Interruption of ART over periods of 3 months or greater are referred to as ART interruption/discontinuation and modelled explicitly. ART interruption/discontinuation is usually concomitant with disengagement from clinic attendance. Average adherence in each 3 month period for an individual is determined from the underlying tendency to adhere (which is a lifelong value for the individual, unless changed as a result of an adherence intervention) with within-person period-to-period variability. Each patient thus has a certain higher or lower tendency to adhere but their actual adherence varies over time, both at random and according to factors such as age, gender, presence of symptoms and experiencing an enhanced adherence intervention as a result of a viral load measured > 1000 copies/mL, as detailed below. Effects of adherence on viral load and resistance acquisition risk are modelled by classifying levels into < 50%, 50-79%, $\geq$ 80%, with effects of ART on viral load suppression being greater the higher the adherence level and the resistance acquisition risk being highest in the 50%-79% category. We do not distinguish between patterns of adherence at a level more granular than the 3 monthly average level and hence cannot explicitly take into account the specific pattern within the 3 month period, which could be important (e.g. whether 80% adherence consists of missing drug one day in every five or a 1 week interruption in every 5 weeks). Thus the adherence level in each period should be conceived of as conveying the degree to which the pattern of adherence means that drug levels are maintained at intended therapeutic levels, rather than simply the average adherence over the period. The distribution of adherence levels was primarily determined by the adherence levels required for the model outputs to mimic observed data. This includes data on rates of resistance development and virologic failure and also data on the proportion of patients at first virologic failure who have no resistance mutations present[4-19].

Consistent with evidence that people tend to have different tendencies to adhere, adherence is modelled using two components. Each patient has a certain greater or lesser tendency to adhere (*adhav,* measured on a scale of 0-100%) but, as described above, their actual adherence in a given period varies over time. Adherence in a given 3 month period is referred to as *adh{t}*. *adhvar* is the standard deviation representing the within-person period-to-period variability over time. Thus, adherence at any one period is initially determined as follows (although with modifications explained below):- adh(t) = *adhav* + Normal(0,$adhvar^2$). An example of how the the distribution of the values of *adhav* and *adhvar* are specified as follows and as illustrated in Figure S3. We consider a range of such patterns and sample at random from the distribution of *adh_pattern*. The different adherence profiles from which we sample are as follows:

A

1% probability	*adhav* = 10%	*adhvar* = 20%
1% probability	*adhav* = 79%	*adhvar* = 20%
18% probability	*adhav* = 95%	*adhvar* = 5%
80% probability	*adhav* = 95%	*adhvar* = 2%

B

3% probability	*adhav* = 10%	*adhvar* = 20%
2% probability	*adhav* = 79%	*adhvar* = 20%
15% probability	*adhav* = 95%	*adhvar* = 5%
80% probability	*adhav* = 95%	*adhvar* = 2%

C

3% probability	*adhav* = 10%	*adhvar* = 20%
3% probability	*adhav* = 79%	*adhvar* = 20%
14% probability	*adhav* = 90%	*adhvar* = 6%
80% probability	*adhav* = 95%	*adhvar* = 5%

D

5% probability	*adhav* = 10%	*adhvar* = 20%
7% probability	*adhav* = 79%	*adhvar* = 20%
8% probability	*adhav* = 90%	*adhvar* = 6%
80% probability	*adhav* = 95%	*adhvar* = 5%

E

5% probability	*adhav* = 10%	*adhvar* = 20%
10% probability	*adhav* = 79%	*adhvar* = 20%
85% probability	*adhav* = 95%	*adhvar* = 2%

F

5% probability	*adhav* = 10%	*adhvar* = 20%
10% probability	*adhav* = 79%	*adhvar* = 20%
27% probability	*adhav* = 90%	*adhvar* = 6%
38% probability	*adhav* = 90%	*adhvar* = 5%
20% probability	*adhav* = 95%	*adhvar* = 5%

G

15% probability	*adhav* = 10%	*adhvar* = 20%
15% probability	*adhav* = 70%	*adhvar* = 20%
50% probability	*adhav* = 90%	*adhvar* = 6%
20% probability	*adhav* = 95%	*adhvar* = 5%

H

20% probability	*adhav* = 10%	*adhvar* = 20%
20% probability	*adhav* = 79%	*adhvar* = 20%
40% probability	*adhav* = 90%	*adhvar* = 6%
20% probability	*adhav* = 95%	*adhvar* = 5%

I

30% probability	*adhav* = 10%	*adhvar* = 20%
30% probability	*adhav* = 60%	*adhvar* = 20%
10% probability	*adhav* = 70%	*adhvar* = 6%
30% probability	*adhav* = 90%	*adhvar* = 5%

Figure S3. Illustration of adherence pattern assumptions. This is for adherence pattern F. 5% of the population have the adherence as shown in the top left, 10% as shown in the top right, etc. While adherence is generally high in the majority of people on ART (hence the high proportion of people on ART with viral suppression), most probably experience at least some periods of poorer adherence (e.g.[20]).

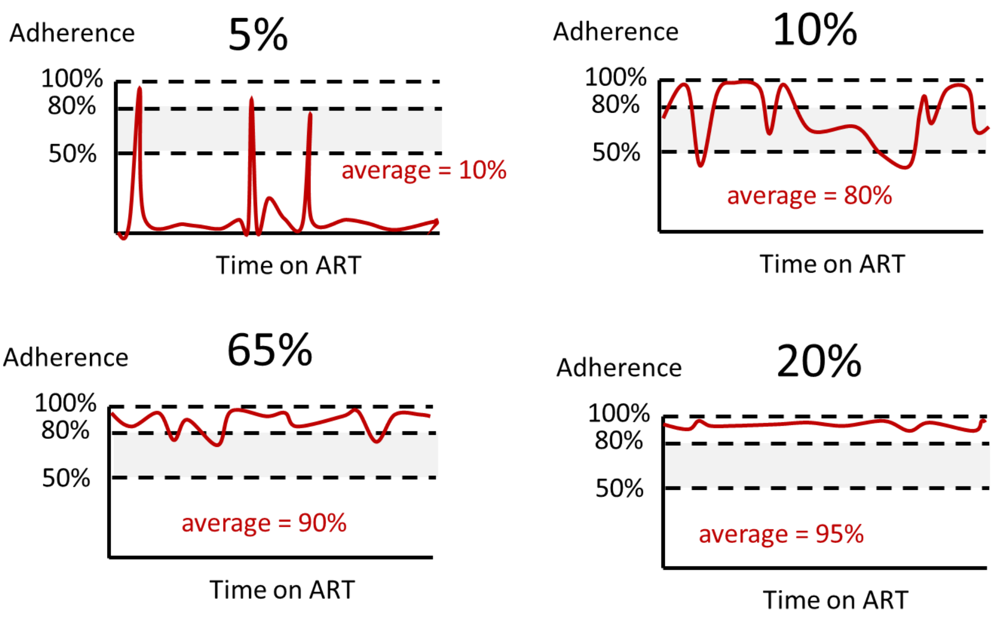

The above describes how we determine the person-specific underlying adherence (*adhav*) and variation in adherence (*advar*). *Adhav* remains constant over a person's lifetime, with the exception that it can increase as a result of an **adherence intervention due to viral load measurement above 1000 cps/mL** (see below), or **when starting 2nd line ART** (due to the fact that there is the emphasis that this is likely the last line of ART available – a last chance to be adherent, albeit that this effect can be counteracted by the lower adherence described below due to toxicity of atazanavir). The increase in *adhav* when starting second line is person-specific and given by 5% x exp(Normal(0, 1)).

Given *adhav* and *adhvar*, the adherence in any one period is determined as follows. First adh(t) = *adhav* + Normal(0,$adhvar^2$). Then adh{t} can be modified according to presence of a drug toxicity, a current WHO stage 4 condition or TB, or gender and age.

Effect of current drug toxicity and current TB or WHO stage 4 condition on adherence

The effect of drug toxicity on adherence is person-specific and given by 5% x exp(Normal(0, 0.3)). In any one 3 month period in which a toxicity is present there is 30% chance of this effect operating. The effect of drug toxicity on adherence is most clearly seen in randomized double-blind trials in which, for example, discontinuation rates are higher for efavirenz-based 1st line compared with a dolutegravir-based regimen [21-22]. During the time of a WHO stage 4 or TB disease, adherence is assume to be reduced by 10%..

Effect of age and gender on adherence

There is an effect of age on adherence, partly evidenced by differences in viral suppression levels, most notably with lower adherence in the 15-20 year age group compared with oler ages [23-28]. Gender is also an influence, as suggested by the higher proportion of women on ART with viral suppression in the PHIA surveys [29].

For men age 15-19 / 20-24 / 25-29, if initially there is adh{t} in a period above 80% there is a 30% / 20% / 10% chance of the adherence being reduced: 65% in two thirds of men and 10% in one third.

For women age 15-19, if initially there is adh{t} in a period above 80% there is a 20% chance of the adherence being reduced: 65% in two thirds of men and 10% in one third.
For women age 20-24 / 25-29 / 30-34 / 35--49 / 50+, if initially there is adh{t} in a period below 80% there is a 10% / 30% / 50% / 80% / 90% chance of the adherence being increased to 90%.

Comparisons between model outputs and data from the literature in Figure 4-10 illustrate the extent to which the model captures various aspects of virologic responses to ART (efavirenz based regimens).

Figure S4. Risk of virologic failure while on ART according to adherence level

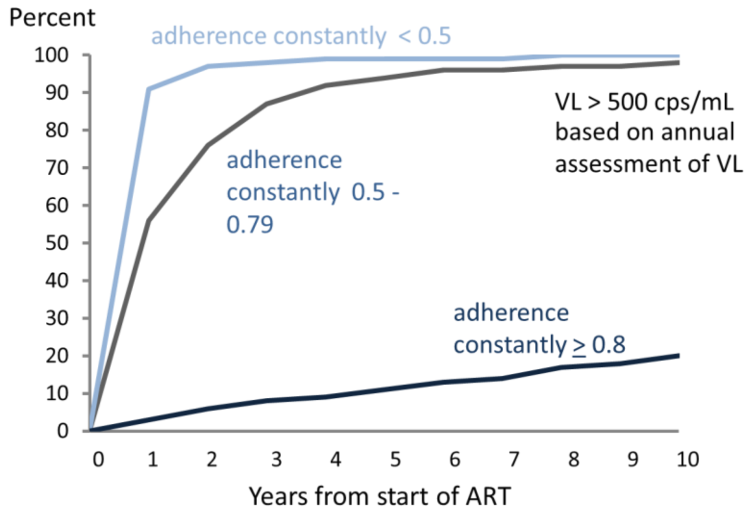

Figure S5. Risk of NNRTI resistance with virologic failure while on ART, according to adherence level

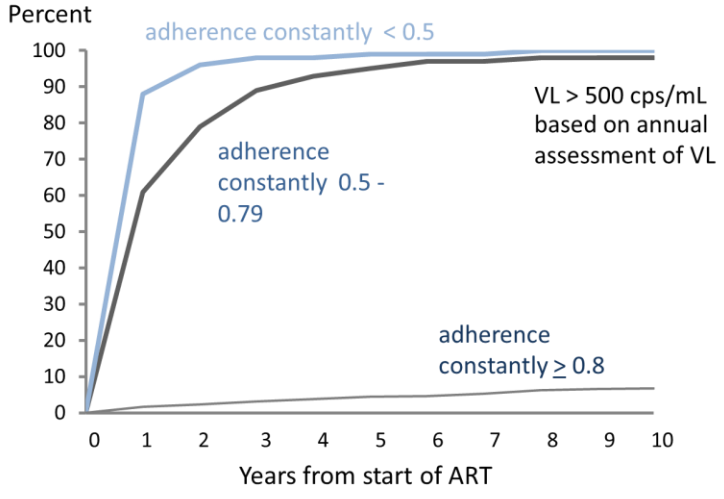

The distribution of adherence over the first year of ART has been compared with data from a large programme in Zambia (see Figure S6; [30]). Viral load suppression at one year from start of ART is shown in Figure S7. These are reconstructed outcomes for all people who have initiated ART in Zimbabwe (the overall mean CD4 count at initiation is 145 /mm^3). Figure S8 and Figure S9 compare Kaplan-Meier estimates of time to virologic failure and resistance, respectively, between the model and observed data, in the latter case from the UK due to the

lack of data from sub-Saharan Africa (although noting that a substantial minority of people in the UK database originate from sub-Saharan Africa). Figure S10 illustrates the proportion of people with resistance (amongst those on ART with non-suppressed viral load) and corresponds to estimates from the large WHO resistance surveillance.

Figure S6. Distribution of average adherence level over first year of ART (for those on ART at 1 year)[30].

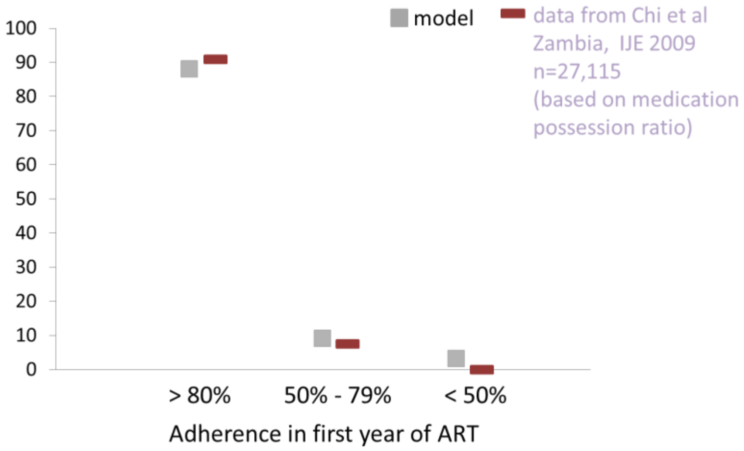

Figure S7. (a) Percent of people alive at given time points from start of ART who have viral load suppression and (b) percent of people alive and on ART at given time points from start of ART who have viral load suppression[31].

(a)

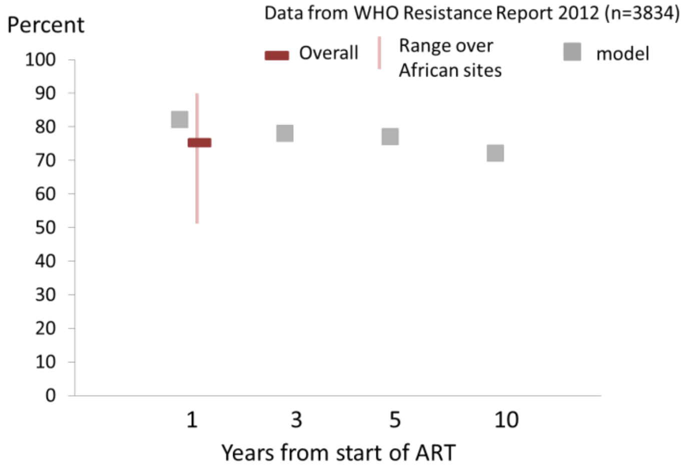

(b)

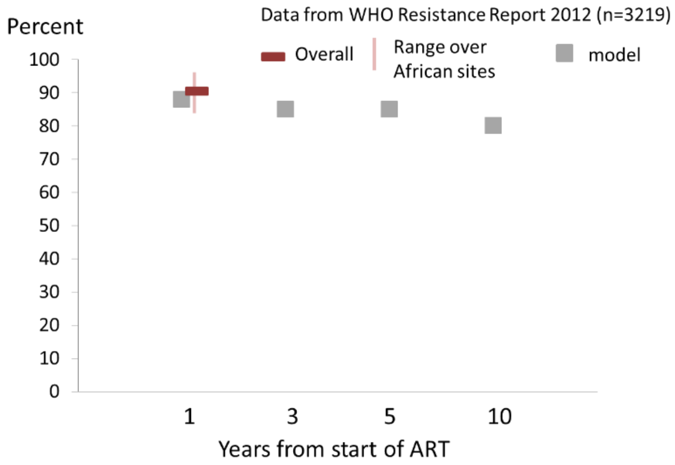

Figure S8 Kaplan Meier estimates of risk of virologic failure while on ART, by time from start of ART[2].

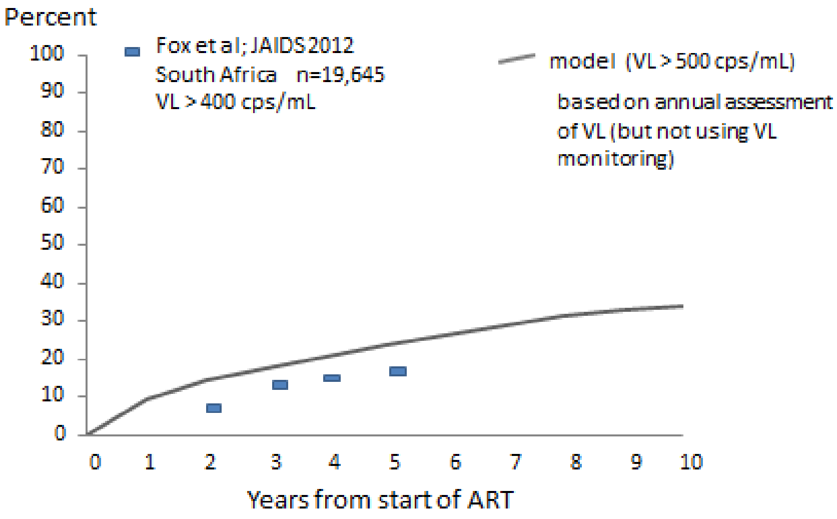

Figure S9. Kaplan Meier estimates of risk of NNRTI resistance with virologic failure while on ART, by time from start of ART[32].

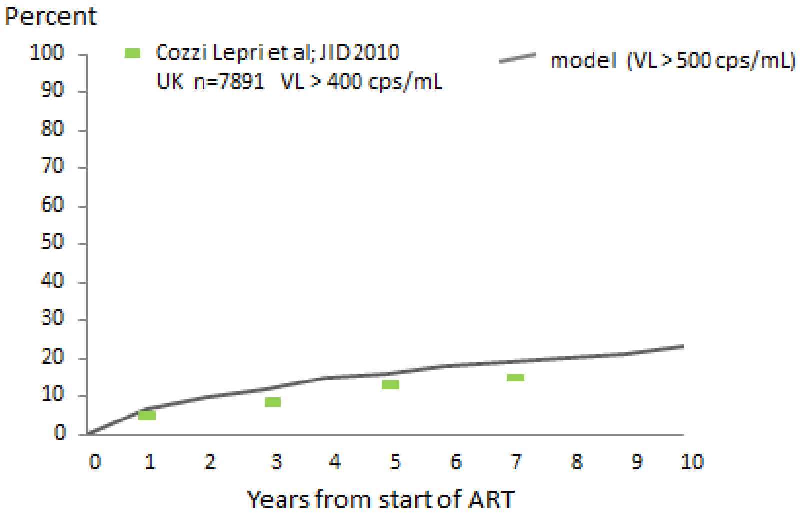

Figure S10. Of people with viral load > 500 at 1 year from start of ART, percent who have NNRTI drug resistance[31].

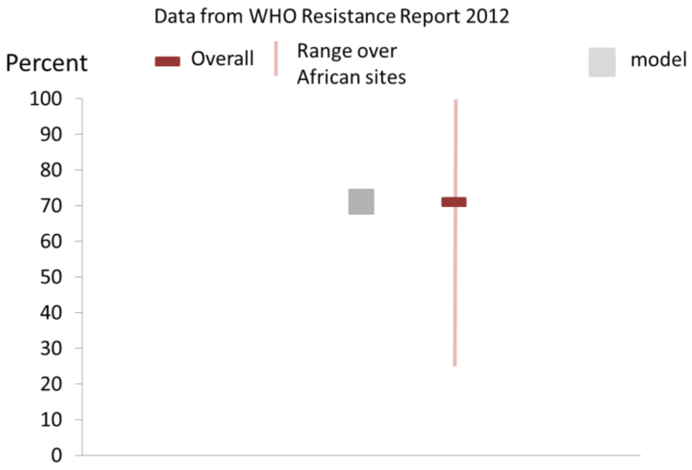

Effective adherence

We also considered the concept of *effective* adherence, which reflects predicted adequacy of drug levels, whereby for those on regimens that do not include an NNRTI the effective adherence is as the adherence itself, but for those on NNRTI-containing regimens the effective adherence is the adherence + *add_eff_adh_nnrti* (base value Log normal(ln 0.10, 0.30)), reflecting the long half life of NNRTI drugs[33] which is an advantage as it means such regimens are more forgiving of periods of poor adherence[5-7, 12, 34-37]. Additionally,-it is assumed that patients on ART are susceptible to occasional (rate 0.02 per 3-months severe temporary drops in drug level (i.e. effective adherence level), leaving them susceptible to viral rebound (but with low risk of resistance as the effective adherence drop is so profound). This phenomenon is assumed to be 100 times more frequent among those on protease inhibitor regimens than in those on other regimens. This

latter assumption is the only plausible means (at least within our model framework) to explain why virologic failure occurring on boosted protease inhibitor regimens often occurs in the absence of resistance[38].

Effect of viral load measurement above 1000 cps/mL on adherence

As mentioned, adherence can be affected by experience of an enhanced adherence intervention after initial measurement of viral load > 1000 copies/mL which is assumed to lead to an increase in adherence in 70% of people, consistent with data showing that a significant proportion of people with measured viral load > 1000 copies/mL who undergo an adherence intervention subsequently achieve viral suppression without a change in ART [10,11,39,40] and broadly consistent with a meta-analysis[41]. Although the appropriate duration to assume for this effect is uncertain[11], the impact of adherence interventions has often been shown to diminish with time[42]. Based on this overall body of data, we assume that the adherence intervention is effective only the first time it is performed and that for 40% the effect is permanent (i.e. 70% x 40%= 28% of those with a viral load >1000; in this case the value of *adhav* is reduced from this point), but that in the remaining 60% (i.e. 70% x 60% = 42% of those with viral load>1000) it lasts only 6 months.

ART interruption / discontinuation

People can interrupt ART, and this may be due to not continuing with clinic visits (disengagement, modelled as simultaneous interruption and loss to clinic follow up) but ART can be interrupted also in those still attending clinical visits. The basic rate of interruption due to patient factors (refered to as *rate_int_choice*, although recognising that this is often not a free choice) is greater in people with current toxicity (2-fold) and those with a greater tendency to be non-adherent (1.5-fold if adherence average *adhav* 50 – 79% and 2-fold if adherence average *adhav* < 50%). In a systematic review, drug toxicity, adverse events and side effects have been found to be the most commonly given reasons for drug discontinuation[43].

The rate of interruption also reduces with time on ART, decreasing after 1 years[44-46]. If adherence average (*adhav*) $\geq$ 80% then the chance that interruption coincides with interrupting/stopping visits to the clinic is equal to *prob_lost_art*; if 50 <= *adhav* < 80% then *prob_lost_art* is multiplied by 1.5, if *adhav* < 50% then *prob_lost_art* is multiplied by 2. This is due to an assumption that factors leading to poor adherence are also likely to be associated with interruption. The rate of interruption and disengagement with care is likely to vary by setting. Figure S11 shows a comparison between modelled and observed (from a study by Kranzer et al[44]. Kaplan Meier estimates of the percent of people having interrupted or discontinued ART by time from ART initiation.

Figure S11. Percent who have interrupted or discontinued ART by time from initiation[44].

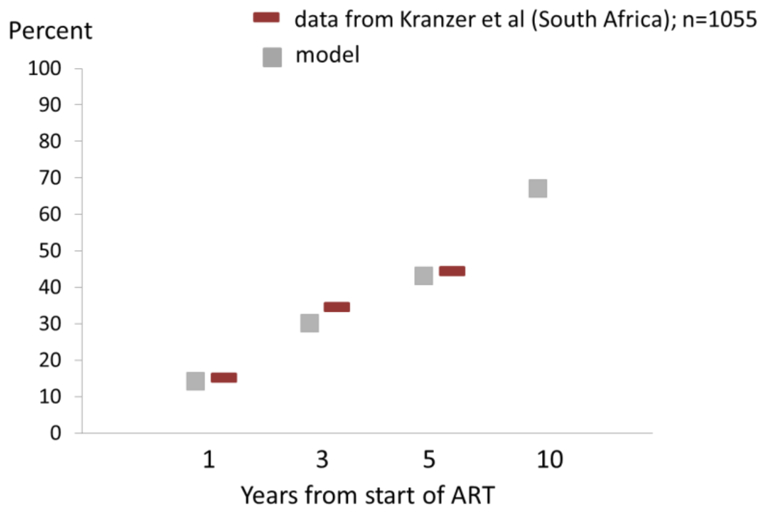

Interruption of ART without clinic/clinician being aware

It is known that in some instances people on ART have such poor adherence that they have in fact interrupted or stopped ART entirely but, in the same way that the clinic is not always aware of the true adherence level, they are also not always aware when the person has completely interrupted ART. This means that the clinic may think a patient is virologically failing, because viral load is high, when in fact this is due to interruption rather than resistance. This can be seen from studies on people with virologic failure in which a proportion have no identified resistance mutations[8,10,47]. Thus, when a person interrupts ART (but remains under care) we introduce a variable that indicates whether the clinic is unaware. *clinic_not_aw_int_frac* (base value Beta (6,4), median=0.61). This distribution was chosen to produce realistic model outputs for the proportion of people with virological failure who have resistance. If a patient has interrupted ART with the clinic unaware then not only is the patient (wrongly) classified (by the clinic) as virologically failing (if viral load has been measured), but a switch to second line can occur. Figure S12 compares the proportion of people with resistance between our model and WHO survey data.

Re-initiation of ART after interrupting in patients still under clinic follow-up

For patients who have interrupted ART due to choice but are still under clinic follow-up, the probability of restarting ART per 3 months in the base model is *rate_restart*. This probability is increased 3-fold if a new WHO 3 condition has occurred at t-1, and 5-fold if a new WHO 4 condition has occurred at t-1 since occurrence of clinical disease in a person seen at clinic is likely to prompt ART re-initiation. This will vary by setting but is informed by studies showing that of people who have initiated ART who are still seen at clinic a very high proportion are on ART at 12 months from start of ART[48]. Kranzer et al found a rate of restarting ART amongst those that interrupted or discontinued of 21 per 100 person-years but this figure is an overall figure which includes in the denominator those who are not attending the clinic (loss to follow-up and return to care are described below)[44]. The equivalent figure, produced as an output from the model is 19 per 100 person-years.

Interruption due to drug stock-outs

The basic rate of interruption due to interruption of the drug supply is *prob_supply_interrupted* per 3 months. This will vary over time and by setting but we assume low rates in current and future years (0.003 per 3 months per person). For patients who have interrupted ART due to interruption of supply the probability of restarting ART per 3 months is *prob_supply_resumed*[48].

Loss to follow-up while off ART (for reasons apart from drug stock-outs)

The probability per 3 months of interrupting/stopping clinic visits (i.e. being lost to follow-up) is *rate_lost* if adherence average *adhav* ≥ 80%. This is increased by 1.5 fold if 50% ≤ *adhav* < 80% and by 2-fold if *adhav* < 50%. This high rate is informed by the fact that low numbers of people attending clinics after having been initiated on ART are not still on ART (e.g.[31]). Interruption of ART and loss to follow-up are assumed correlated with the underlying tendency to adhere when on ART because we assume that the same underlying social, practical and economic factors will be an underlying cause of these behaviours.

For people lost to follow-up who are asymptomatic, the probability of returning to clinic per 3 months is *rate_return* if adherence average *adhav* ≥ 80%. This is decreased by 2-fold if 50% ≤ *adhav* < 80% and by 3-fold if *adhav* < 50%. If a person develops a new WHO 3 or 4 event then they are assumed to return to the clinic with probability 1. As mentioned above, this leads to an overall rate of restarting of ART after interruption (including having been loss to follow-up in many cases) consistent with the estimates from South Africa from Kranzer et al, although these will vary by setting[2,35,49].

Figure S12. Status at 1 year from start of ART. Data is from WHO Drug Resistance Surveillance Report (2012)[31].

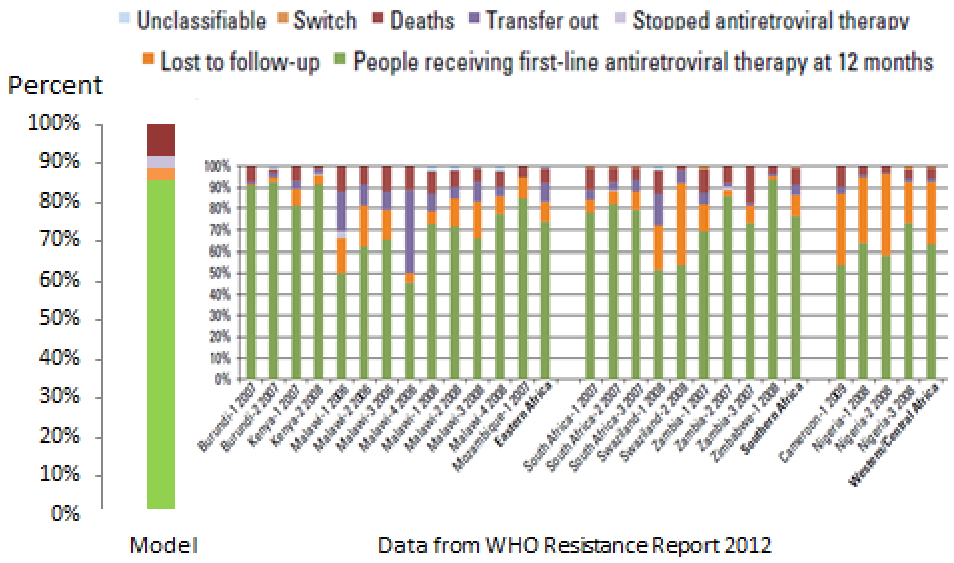

Effect of ART on viral load, CD4 count, resistance development and drug toxicity

This section describes the determination of updated viral load, CD4 count, and acquisition of new resistance mutations in a given time period for people on ART. The updated viral load, CD4 count and risk of new resistance mutations appearing all depend on the effective adherence in the previous and current period, the number of active drugs (*nactive(t-1)*) and the current viral load, as well as the time period from the last time ART was started or restarted. The values of viral load, CD4 count, and resistance mutation risk for any combination of these factors are given in Table S1-S3 below. The rationale behind this approach and how the specific values in the table were chosen is explained below. The choice of values is directly informed by studies in this area and by comparison of model outputs with data. For the new resistance mutation risk, the number in the table is multiplied by the viral load (mean of values at t-1 and t) to give a value for the variable *newmut*, which is used when assessing whether a new mutation or mutations have arisen (see below).

Number of active drugs

We use the concept of the number of drugs that are active, based on presence of resistance mutations to the drugs being used. The level of resistance is determined by the presence of drug resistance mutations, with a given set of mutations being translated into a level of resistance to a given drug on a scale of 0 to 1 in the same way as is done for common resistance interpretation systems. The activity level of a drug is then calculated as 1 minus the level of resistance to the drug. The ability of the number of active drugs, or the genotypic sensitivity score, to predict the viral load outcome is well established[50], and the concept of using a genotypic score to define "optimised background therapy" has been common to the design of several trials in treatment experienced patients (e.g. [51]). This is the basic concept but note that below we explain consider that drugs, such as boosted PIs, can have higher potency (since they can virtually sustain viral suppression alone) and thus contribute a value greater than 1.

Classification of adherence levels

While we model the adherence level for each individual at each three month time period as a value between 0 and 100%, to determine the viral load, CD4 count and resistance risk, as noted above, we classify adherence into three levels. This is the simplest approach that allows inclusion of the fact that the relationship between adherence and resistance risk is not linear, since the risk of resistance tends to be lower when the adherence is either low or high, and the risk of resistance is highest when adherence is moderate, allowing enough replication for mutations to be selected for and enough drug present to allow selection of virus with resistance mutations[5,34,52].

As mentioned, the cut-offs used to define the three adherence levels are 50% and 80%. Adherence-resistance and adherence-viral load relationships differ by regimen type and even specific regimen within a class and any overall breakdown into groups is necessarily a simplification. A cut off of 80% is chosen as the upper level as (unlike for unboosted PI regimens) at adherence levels of at least 80%, NNRTI and boosted PI regimens are likely to have maximal or close to maximal effects on viral load and minimal risk of resistance selection[37]. Actual risk of resistance probably depends on the pattern of adherence, not just the average over a three month period, so that a treatment interruption of over 1 week during the three month period, while maintaining an overall average adherence of 80%, could lead to a higher level of risk of resistance emergence than a situation in which the adherence was more uniform over the period[53], although in people who have ongoing viral suppression NNRTI regimens seem to be generally robust to even relatively low levels of adherence [35-37, 54]. A level below 50% is one that that has been associated with raised risk of detectable viral load[53,55].

Determination of viral load, CD4 count and risk of resistance in people on ART

Viral load, CD4 count and risk of resistance in the first 3 months after (re-)starting ART

Table S1 shows how the viral load, CD4 count and risk of resistance is determined for people in the first 3 months after starting ART or re-starting ART after an interruption of at least 3 months. Since in this early period on ART, the viral load will depend on the initial value the updated viral load is given as a reduction from the pre-ART maximum viral load. If the number of active drugs is three or more then at a high adherence level (above 0.8) the mean viral load change from the pre-ART maximum is 3 log copies/mL. To reflect the fact that there is variability in the response[56], the value for a given person is sampled from a Normal distribution with standard deviation 0.5. This viral load response diminishes both with decreasing number of active drugs in the regimen being started (which is informed by data from studies relating GSS to virologic outcome, as well as by studies of mono and dual therapy regimens[50, 57-63]. The viral load response also diminishes with decreasing level of adherence (see Figure 513 and for example Genberg et al[53]. As is well established, the CD4 count response generally mirrors the viral load response, although with very low numbers of active drugs and low

adherence there is a mean decrease in CD4 count and still a small decrease in viral load from the maximum. Note that we do not incorporate the known more rapid decline in viral load seen with integrase inhibitors.

Figure S13. Model output: of people on ART, percent with current VL >500 according to current adherence. Comparison with data from Genberg at el on electronic monitoring-based adherence measures[53].

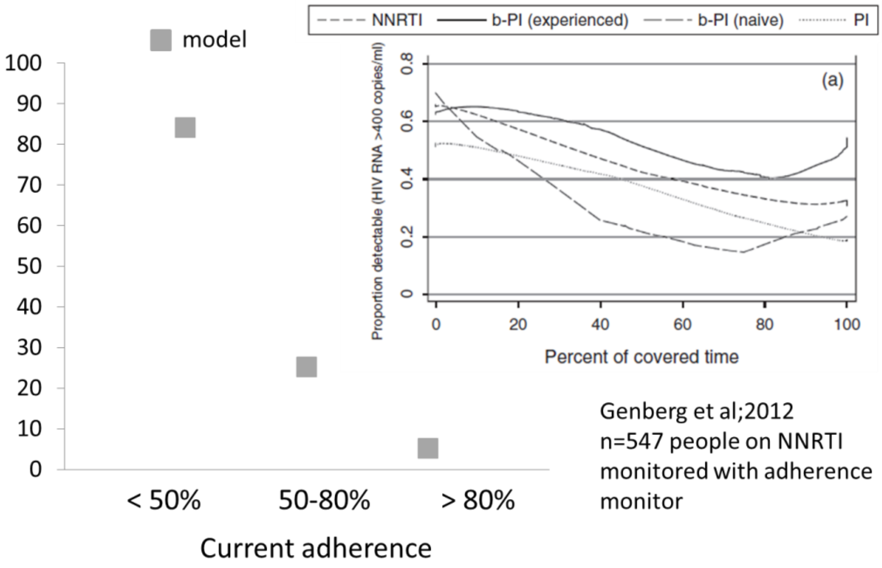

Regarding the risk of new drug resistant mutations arising, Tables S1-S3 provide a number for “new mutation risk” that is multiplied by the viral load (mean of values at t-1 and t) to give a probability used when assessing whether a new mutation(s) has/have arisen. Values of the new mutations risk have been chosen in conjunction with the translation of presence of mutations into reduced drug activity to provide estimates of resistance accumulation consistent with those observed in clinical practice[19 64-70].
Risk of new resistance mutations arising increases with decreasing number of active drugs, reflecting the known greater risk of resistance with regimens less able to suppress viral replication, most clearly seen in the fact that early mono and dual therapy regimens were highly susceptible to resistance development[49-51]. At low adherence levels, the risk of resistance development is generally low regardless of the number of active drugs, as drug selection pressure is low. However, for those on NNRTI regimens the new resistance mutation risk is assumed to be that for the effective adherence category of 50 – 80% (i.e. maximal) even if the effective adherence is below 50%, reflecting the fact that NNRTI resistance develops easily, even when drug exposure is very low[5,6].

Viral load, CD4 count and risk of resistance between 3-6 months from (re-)starting ART

For the period 3-6 months from (re-)start of ART (Table S2; to reduce the table content we do not provide the matrices of values for the resistance risk or CD4 count, only for the viral load (the full table is available in Cambiano et al 2014 [71]). We consider the adherence in both the current and previous 3 month period, since the likelihood of reaching viral suppression by 6 months will depend on adherence throughout the whole 6 month period from start of ART, although the adherence in the current period is assumed to be the stronger factor. By 6 months after starting ART, those on 3 or more active drugs with consistently high adherence generally reach a relatively high level of viral suppression, regardless of pre-ART maximal viral load, so a person’s viral load is no longer given by the change from baseline but the absolute level of viral load which it is likely they have reached. In these optimal conditions of high adherence and maximal active drugs we assume the viral load has a mean value of 0.5 log, again with variability between individuals. Since most viral load

assays have a lower limit of quantification of 40 or 50 copies per mL, it is not actually known what the actual viral load level is, although highly sensitive assays suggest that a proportion of patients reach below 11 copies/mL[72]. At lower numbers of active drugs and lower adherence, the viral load is still related to the maximal pre-ART viral load rather than being an absolute value, as the person's viral load has not become so low that the initial value loses relevance. The viral load response decreases with a lower number of active drugs, lower current adherence, and lower adherence in the previous 3 month period. Values for the viral load response between those known from studies (high level of suppression for 3 active drugs and maximal adherence, and only around 0.5 log viral suppression when adherence is < 0.5 even with three active drugs[61,73] are imputed assuming a monotonic relationship. CD4 count responses again mirror the viral load response, as has been extensively studied in patients with ongoing viraemia on ART[74]. Risk of new resistance mutations again increases with decreasing number of active drugs, if current adherence is in the middle or highest group. The only situation in which risk of new mutations is extremely low is when the number of active drugs is 3 or close to 3 and the current adherence is in the high category.

Viral load, CD4 count and risk of resistance after 6 months of (re-)starting ART

Table S3 shows how the viral load, CD4 count and risk of resistance is determined for the situation where a person has been on ART for more than 6 months and the viral load is suppressed or partially suppressed (< 4 log copies/mL). These values are similar to those used for the period 3-6 months from start of ART except that there is assumed to dependence on the adherence in the current 3 month period only.

The situation where the viral load is above 4 log copies /mL, 10,000 copies/mL is treated the same as that in the period 3-6 months from start of ART (described above), with adherence in the current and previous period having some influence.

Variable patient-specific tendency for CD4 count rise on ART

There is variability in the tendency for the CD4 count to rise on ART, for a given level of viral load suppression. For scenarios in the above tables (S1 – S3) in which the CD4 count change is positive the CD4 count change is multiplied by this patient-specific factor (i.e. it is fixed for each patient), which is given by sampling for each patient from Exp (N(0, (*sd_patient_cd4_rise_art*)^2) where sd_patient_cd4_rise_art = 0.2. To reflect the fact that the rate of CD4 count increase on ART tends to diminish with time, for those with patient-specific factor determining the CD4 count rise on ART > 1, this factor is divided by 1.25 after 1 year of continuous treatment, by 3 after 3 years of continuous treatment and by 10 after 5 years. In addition there is a dependence of CD4 increase on current CD4 level with a multiplying factor of 0.85-fold if the CD4 count is 100-200 and 0.7-fold if > 200. Comparisons of resultant outputs with observed data are described below.

Accelerated rate of CD4 count loss if PI not present in regimen

The rate of change in CD4 count in people on failing regimens is largely based on data from the PLATO collaboration, for which patients were mainly on regimens containing a PI[74]. If the regimen does not contain a PI the change in CD4 count per 3 months is modified (in the base model) by *poorer_cd4_rise_on_failing_nnrti* (= -6 /mm^3). This applies regardless of viral load level, so PIs are assumed to lead to a more beneficial CD4 count change than NNRTIs[74]. We assume in 50% of setting scenarios that this applies also for dolutegravir and in 50% that it does not (determined by *poorer_cd4_rise_on_fail_nn_ii*).

Variability in individual (underlying) CD4 counts for people on ART

Once the mean of the underlying CD4 count is obtained as described above for people on ART, to obtain the CD4 count, variability (*sd_cd4* = 1.2) is added on the square root scale. The estimate was based on unpublished analyses

The ongoing CD4 count increases in people with viral suppression are informed by Mocroft et al 2007 [75]. CD4 count As a result of these assumptions, model outputs for mean changes in CD4 count from start of (efavirenz-based) ART in people with ongoing viral suppression at years 1, 3, 5, 10 are 200, 338, 442 and 633. Amongst people starting ART with CD4 < 100 at start of ART, the proportion of people with a CD4 count above 500/mm3 by 5 years from start of ART is 62%. Similar figures for people with baseline CD4 count 100-200, and 200-300 are 75% and 78% respectively. In comparison, Bishop et al report CD4 count changes of 206, 278, 419, 509 at 1, 3, 5 and 10 years respectively [76]. Geng et al report a CD4 count increase of 365 /mm3 in people in southern Africa starting with a CD4 count of 150/mm3 [77]. At 5 years from start of ART O'Connor et al report a proportion with CD4 count > 500 of 60% for people starting ART with CD4 count < 100 [78]. In the Single trial the CD4 count rise at 1 year was 208/mm^3 and at 3 years was 332/mm^3 [21-22].

Each person has a maximum attainable CD4 count given by sampling from exp(Normal(7.3, 0.25^2)).

Table S1. Viral load (mean change from viral load max), CD4 count change (mean change between t-1 and t), and new mutation risk in first 3 months. For 0 active drugs, these are the changes regardless of time from start of ART. For viral load this is the mean of a Normal distribution with standard deviation 0.2, from which the patient's value/change is sampled. For the CD4 count patients vary in their underlying propensity for CD4 rise on ART (given by sampling from lognormal(1,0.5^2) and the CD4 count change given here is multiplied by this factor. For the new mutation risk, this is a number that is multiplied by the viral load (mean of values at t-1 and t). The resulting probability is used when assessing whether a new mutation or mutations have arisen.

	Effective adherence between t-1 & t	Number of active drugs												
		3	2.75	2.5	2.25	2.0	1.75	1.5	1.25	1	0.75	0.5	0.25	0
Viral load	≥ 80%	-3.0	-2.6	-2.2	-1.8	-1.5	-1.25	-0.9	-0.8	-0.7	-0.55	-0.4	-0.3	-0.3
(log change	≥ 50%, <80%	-2.0	-1.6	-1.2	-1.1	-0.9	-0.8	-0.6	-0.5	-0.4	-0.25	-0.1	-0.05	-0.1
from vmax)	< 50%	-0.5	-0.4	-0.3	-0.25	-0.2	-0.15	0.0	+0.05	+0.1	+0.1	+0.1	+0.1	0.0
CD4 count	≥ 80%	+50	+45	+40	+35	+30	+25	+20	+17	+13	+10	+5	-2	-15
change	≥ 50%, <80%	+30	+30	+23	+20	+15	+13	+10	+8	+5	+3	0	-7	-17
(t-1 to t)	< 50%	+5	+4	+3	+2	+1	-1	-3	-6	-10	-11	-12	-13	-18
New mutation	≥ 80%	0.002	0.01	0.03	0.05	0.1	0.15	0.2	0.3	0.4	0.45	0.5	0.5	0.5
Risk	≥ 50%, <80%	0.15	0.15	0.2	0.25	0.3	0.3	0.3	0.35	0.4	0.45	0.5	0.5	0.5
(x log viral load)	< 50%*	0.15	0.15	0.2	0.25	0.3	0.3	0.3	0.35	0.4	0.45	0.5	0.5	0.5
	< 50%**	0.05	0.05	0.05	0.05	0.05	0.05	0.05	0.05	0.05	0.05	0.05	0.05	0.05

* for NNRTI containing regimen, ** for boosted PI containing regimen.

Table S2. Summary of viral load (mean <u>absolute value</u> or mean change from viral load max) between 3-6 months, and after 6 months if viral load at t-1 > 4 logs. This is the mean of a Normal distribution with standard deviation 0.2, from which the patient's value/change is sampled.

Effective adherence between t-2 & t-1	Effective adherence between t-1 & t	Number of active drugs											
		3	2.75	2.5	2.25	2.0	1.75	1.5	1.25	1	0.75	0.5	0.25
≥ 80%	≥ 80%	<u>0.5</u>	<u>0.8</u>	<u>1.2</u>	<u>1.4</u>	<u>2.0</u>	<u>2.7</u>	-1.7	-1.15	-0.9	-0.75	-0.6	-0.4
≥ 50%, <80%	≥ 80%	<u>1.2</u>	<u>1.2</u>	<u>1.2</u>	<u>1.4</u>	-2.0	-1.6	-1.2	-1.05	-0.9	-0.7	-0.5	-0.35
< 50%	≥ 80%	<u>1.2</u>	<u>1.2</u>	<u>1.2</u>	<u>1.4</u>	-2.0	-1.6	-1.2	-1.0	-0.9	-0.7	-0.5	-0.2
≥ 80%	≥ 50%, <80%	<u>1.2</u>	1.6	<u>1.8</u>	<u>2.2</u>	<u>2.4</u>	-2.4	-1.5	-0.9	-0.7	-0.55	-0.4	-0.3
≥ 50%, <80%	≥ 50%, <80%	<u>2.5</u>	<u>2.5</u>	<u>2.5</u>	<u>2.5</u>	-1.2	-1.1	-0.8	-0.65	-0.5	-0.35	-0.2	-0.05
< 50%	≥ 50%, <80%	-2.0	-1.8	-1.5	-1.35	-1.2	-1.1	-0.8	-0.65	-0.5	-0.2	-0.2	-0.05
≥ 80%	< 50%	-0.5	-0.4	-0.3	-0.25	-0.2	-0.15	-0.10	-0.05	+0.0	+0.0	+0.0	+0.0
≥ 50%, <80%	< 50%	-0.5	-0.4	-0.3	-0.25	-0.2	-0.15	-0.10	-0.05	+0.0	+0.0	+0.0	+0.0
< 50%	< 50%	-0.5	-0.4	-0.3	-0.25	-0.2	-0.15	-0.10	-0.05	+0.0	+0.0	+0.0	+0.0

Table S3. Summary of viral load (mean change from viral load max), CD4 count change (mean change between t-1 and t), and new mutation risk after 6 months, where viral load at t-1 < 4 logs. For viral load this is the mean of a Normal distribution with standard deviation 0.2, from which the patient's value/change is sampled. For the CD4 count patients vary in their underlying propensity for CD4 rise on ART (given by sampling from lognormal(1,0.5^2) and the CD4 count change given here is multiplied by this factor. For the new mutation number, this is a number that is multiplied by the viral load (mean of values at t-1 and t). The resulting probability is used when assessing whether a new mutation or mutations have arisen.

	Effective adherence between t-1 & t	Number of active drugs											
		3	2.75	2.5	2.25	2.0	1.75	1.5	1.25	1	0.75	0.5	0.25
Viral load (absolute value or log change from vmax)	≥ 80%	0.5	0.0	1.2	1.6	-2.5	-2.0	-1.4	-1.15	-0.9	-0.75	-0.6	-0.3
	≥ 50%, <80%	1.2	1.2	1.2	1.4	-1.2	-1.0	-0.7	-0.6	-0.5	-0.4	-0.3	-0.1
	< 50%	-0.5	-0.4	-0.3	-0.25	-0.2	-0.2	-0.1	-0.1	-0.1	-0.1	-0.1	-0.0
CD4 count Change (t-1 to t)	≥ 80%	+30	+28	+25	+23	+21	+19	+3	-5	-9	-10.5	-12	-12
	≥ 50%, <80%	+15	+13	+10	+8	-4.5	-7.5	-10	-12	-13	-14	-15	-15
	< 50%	-13	-14	-15	-15.5	-16	-16.5	-17	-17	-18	-17	-17	-17
New mutation risk (x log viral load)	≥ 80%	0.002	0.01	0.03	0.08	0.10	0.15	0.2	0.3	0.4	0.45	0.5	0.5
	≥ 50%, <80%	0.15	0.18	0.2	0.25	0.3	0.3	0.3	0.35	0.4	0.45	0.5	0.5
	< 50%*	0.15	0.18	0.2	0.25	0.3	0.3	0.3	0.35	0.4	0.45	0.5	0.5
	< 50%**	0.05	0.05	0.05	0.05	0.05	0.05	0.05	0.05	0.05	0.05	0.05	0.05

* for NNRTI containing regimen, ** for boosted PI and dolutegravir containing regimen.

Viral load and CD4 count changes during ART interruption

Viral load returns to previous maximum viral load (vmax) in 3 months and adopts natural history changes thereafter.

CD4 rate of decline returns to natural history changes (ie those in ART naïve patients) after 9 months, unless the count remains > 200 above the CD4 nadir

Rate of CD4 count decline depends on current viral load. c(t) is the CD4 count at time t, cmin(t) is the CD4 count nadir measured by time t and cc(t-1) is the change in CD4 count from t-1 to t.

if time off ART = 3 months or if time off ART > 3 months and CD4 in previous period is > 300 above the minimum CD4 count to date

v(t) = vmax(t-1)
if v(t) $\geq$ 5 then cc(t-1) = Normal (-200,10^2)
if 4.5 <= v(t) < 5 then cc(t-1) = Normal (-160,10^2)
if v(t) < 4.5 then cc(t-1) = Normal (-120,10^2)

If this leads to c(t) < cmin(t) (CD4 nadir) then c(t) is set to cmin(t)
if time off ART = 6 months:-

if v(t) $\geq$ 5 then cc(t-1) = Normal (-100,10^2)
if 4.5 <= v(t) < 5 then cc(t-1) = Normal (-90,10^2)
if v(t) < 4.5 then cc(t-1) = Normal (-80,10^2)

if time off ART = 9 months:-

if v(t) $\geq$ 5 then cc(t-1) = Normal (-80,10^2)
if 4.5 <= v(t) < 5 then cc(t-1) = Normal (-70,10^2)
if v(t) < 4.5 then cc(t-1) = Normal (-60,10^2)

This is broadly based on evidence from a number of analyses of the effects of ART interruption (e.g. [79-82])

Incidence of new current toxicity and continuation of existing toxicity
Toxicities including gastrointestinal symptoms, rash, hepatoxicity, CNS toxicity, lipodystrophy, hypersensitvity reaction, peripheral neuropathy and nephrolithiasis can occur with certain probability on certain specific drugs (Table S4). These probabilities are based broadly on evidence from trials and cohort studies, although there are no common definitions for some conditions which complicates this.

Table S4. Risk of development of specific drug toxicities.

Toxicity	Drug	Risk of development per 3 months	Probability of continuation if pre-existing
Nausea	atazanavir	1% (5-fold higher in 1st year)	50%
	zidovudine	3% (5-fold higher in 1st year)	50%
Diarrhoea	atazanavir	1% (2.5-fold higher in 1st year)	50%
Rash	efavirenz	3% (in first 6 months on efavirenz)	
CNS toxicity	efavirenz	10% (if been on efavirenz <1 year)	80% if been on efavirenz <1 year. 90% if been on efavirenz ≥1 year
	dolutegravir	5% (if been on dolutegravir <1 year)	40% if been on dolutegravir <1 year. 90% if been on dolutegravir ≥1 year
Lipodystrophy	zidovudine	1.5%	100%
Anaemia	zidovudine	3% (1.5-fold higher in 1st year)	20%
Headache	zidovudine	10% (1.5-fold higher in 1st year)	40%
Lactic acidosis	zidovudine	0.02%	
Renal dysfunction	tenofovir	0.35%	100%

Switching of drugs due to toxicity

If toxicity is present then we consider in some scenarios that drugs may be switched due to toxicity.

Emergence of specific resistance mutations and their effect on drug activity

newmut (see Table S1 – S3 above) is a probability used to indicate the level of risk of new mutations arising in a given 3 month period. If this chance comes up in a given 3 month period (determined by sampling from the binomial distribution) then the following criteria operate.

Table S5. Risk of acquiring new resistance mutations**.**

Resistance mutation	Probability of arising	Conditions
M184	80%	if on 3TC or FTC
# TAMS increases by 1	20%	if on zidovudine and (not on 3TC nor FTC)
	12%	if on zidovudine and (on 3TC or FTC)
# TAMS increases by 2	1%	if on zidovudine and (not on 3TC nor FTC)
	1%	if on zidovudine and (on 3TC or FTC)
K65	10%	if on tenofovir
Q151	2%	if on zidovudine
K103	60%	If on efavirenz
Y181	10%	If on efavirenz
G190	10%	If on efavirenz
I50L	3%	If on atazanavir
I84V	3%	If on atazanavir
N88	3%	If on atazanavir
primary dolutegravir mutation	3%	if on dolutegravir
secondary dolutegravir mutation	3%	if on dolutegravir

These values are chosen, in conjunction with values of *newmut{t}*, to provide estimates of accumulation of specific classes of mutation consistent with those observed in clinical practice[32, 67, 83]. They reflect a greater propensity for some mutations to arise than others. This probably relates to the ability of the virus to replicate

without the mutations (e.g. probably very low in the presence of 3TC for virus without M184V) as well as the replicative capacity of virus with the mutations.

New resistance to NNRTI arising as a result of ART interruption

It is assumed that due to the long half life of NNRTIs nevirapine and efavirenz, stopping of a regimen containing one of these drugs is associated with a specific probability of an NNRTI resistance mutation arising (see, for example, Fox et al, 2008[4]). The respective probabilities for K103, Y181 and G190 are 1.8%, 0.06% and 0.6%.

Loss of acquired mutations from majority virus

It is assumed that mutations tend to be lost from majority virus with a certain probability from 3 months after stopping to take a drug that selects for that mutation. The probability of losing mutations per 3 months (from 3 months after stopping) is as follows[84-90].

Table S6. Probability of loss of acquired mutations from majority virus per 3 months after stopping drugs selecting for mutation.

M184V	0.8
L74V	0.6
Q151M	0.6
K65R	0.6
TAMS (lose all)	0.4
NNRTI mutations	0.05
Protease mutations	0.2
Dolutegravir mutations	0.2

Mutations are regained in majority virus if a drug selecting for the mutation is again started.

Determination of level of resistance to each drug

Table S7. shows the level of resistance to each drug according to presence of specific resistance mutations.

Table S7. Level of resistance to each drug according to presence of specific resistance mutations.

Resistance mutation	Drug	Level of resistance (1=full resistance)	Condition
M184	3TC or FTC	0.75	
1-2 TAMS	zidovudine	0.5	No 3TC or FTC in regimen
	zidovudine	0.25	3TC or FTC in regimen and ever had M184V
	zidovudine	0.5	3TC or FTC in regimen and never had M184V
2-3 TAMS	tenofovir	0.5	
3-4 TAMS	zidovudine	0.75	No 3TC or FTC in regimen
	zidovudine	0.5	3TC or FTC in regimen and ever had M184V
	zidovudine	0.75	3TC or FTC in regimen and never had M184V
4 or more TAMS	tenofovir	0.75	No 3TC or FTC in regimen, or 3TC in the regimen and never had M184V
	tenofovir	0.5	3TC or FTC in regimen and ever had M184V
5 or more TAMS	zidovudine	1.0	No 3TC or FTC in regimen
	zidovudine	0.75	3TC or FTC in regimen and ever had M184V
	zidovudine	0.75	3TC or FTC in regimen and never had M184V

Q151	3TC or FTC	0.25	
	zidovudine	0.75	
K65	3TC or FTC	0.25	
	tenofovir	0.75	
K103	efavirenz	1.0	
Y181	efavirenz	0.75	
G190	efavirenz	0.75	
I501	atazanavir	1.0	
N88	atazanavir	1.0	
I84	atazanavir	1.0	
1 - 3 of (V32, M46, I54, V82, L90)	atazanavir	0.5	
At least 4 of (V32, M46, I54, V82, L90)	atazanavir	1.0	
primary dolutegravir mutation only	dolutegravir	0.75	
secondary dolutegravir mutation only	dolutegravir	0.25	
primary and secondary dolutegravir mutation	dolutegravir	1.00	

These rules approximately follow the interpretation systems for conversion of mutations present on genotypic resistance test into a predicted level of drug activity (or, equivalently, of resistance; http://hivdb.stanford.edu, http://www.hivfrenchresistance.org/

Calculation of activity level of each drug

For drugs with a potency of 1 the activity level is 1-level of resistance. For ritonavir boosted PIs, which are asigned a potency of 2 it is given by 2 – (2 x level of resistance). Potency is assumed higher due to the ability to induce sustained viral suppression alone. Activity levels of each drug in the regimen are summed to give the total number of active drugs. For dolutegravir the potency is assumed to be 1.5 (the modal value of the distribution) so the activity is 1.5 – (1.5 x level of resistance). We also consider a range of values for the potency of dolutegravir, as described below.

Transmitted resistance: overview

The modelling of transmission of drug resistance is summarized in Figure S14. Readers wishing to understand this in the context of modelling of HV transmission in general should refer to https://www.thelancet.com/cms/10.1016/S2352-3018(17)30190-X/attachment/02742987-df48-4372-8e4a-43888c2ec1e8/mmc1.pdf The presence or not of resistance mutations does not influence the risk of transmission (i.e. virus with resistance mutations present is assumed equally transmissible as virus without such mutations, for a given viral load). The probability that resistance mutations present in majority virus of the source partner are transmitted to the newly infected person is dependent on the specific mutation. Once a resistance mutation is transmitted to the new host it is assumed to have a certain probability of being lost from majority virus over time[91]. Even after being lost from majority virus, it is assumed to remain in minority virus and is selected back as majority virus if an antiretroviral drug selecting for that mutation is initiated. We also consider the possibility of a person who is already infected become super-infected, including with drug resistant HIV[92], although there is assumed to be at most a 20% chance that a person super-infected by a person with HIV resistance then has virus with those resistance mutations as a result.

Figure S14. Overview of modelling of transmission of drug resistance

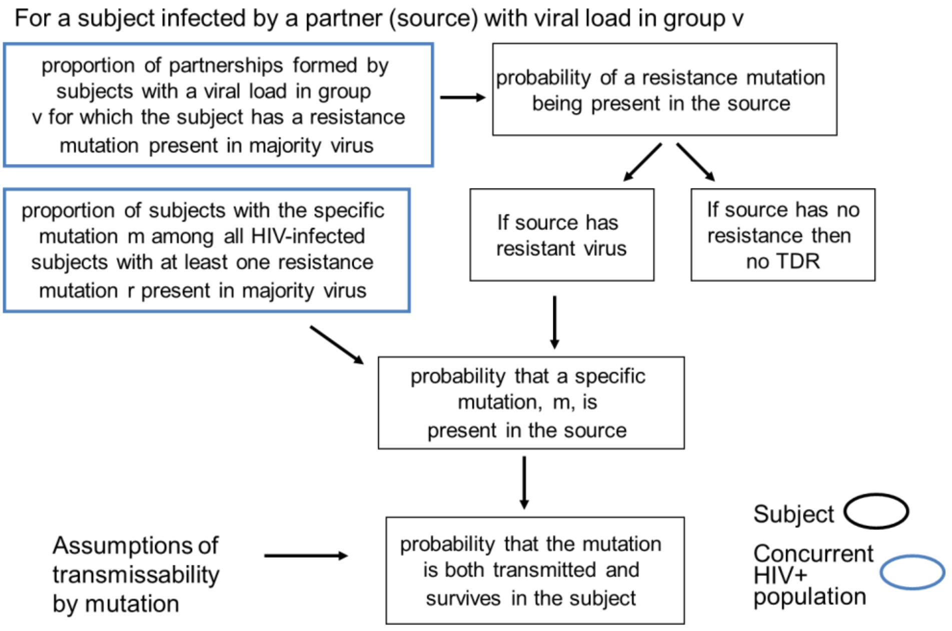

Transmitted resistance: details

The viral load group of the person who infected the subject is known, as indicated above. For a subject infected by a person in viral load group v the probability of a resistance mutation being present in the infected person is given by

$$\frac{\sum_{v,\text{ and mutation present}} L_{(t-1)}^{\text{inf}}}{\sum_{v} L_{(t-1)}^{\text{inf}}}$$

where $\sum_{v,\text{ and mutation present}}$ is the sum over all partnerships had by HIV-infected people in viral load group v for whom a resistance mutation is present in majority virus and $\sum_{v}$ is the sum over all HIV-infected subjects in viral load group v. Realization of whether the subject is infected by a person with at least one resistance mutation in majority virus is determined by sampling from Uniform(0,1).

For subjects infected from a source partner with a resistance mutation, the probability that a specific mutation, m, is present in the source is given by

$$\frac{\sum_{\text{mutation } m \text{ present}} L_{(t-1)}^{\text{inf}}}{\sum_{\text{mutation present } v} L_{(t-1)}^{\text{inf}}}$$

Where $\sum_{\text{mutation } m \text{ present}}$ is the sum over all HIV-infected subjects with mutation m present in majority virus and $\sum_{\text{mutation present}}$ is the sum over all HIV-infected subjects with at least one resistance mutation in majority virus.

If a given resistance mutation, m, is present in the source partner, the probability that the mutation is both transmitted and survives in the subject (i.e. that its presence will affect future response to drugs for which the mutation confers reduced sensitivity) is shown in Table S8.

Table S8. Table of probabilities that for a given mutation present in the source partner the mutation is both transmitted and survives in the subject (based on evidence from studies comparing distribution of resistance mutations between treated and antiretroviral naïve populations; (e.g. [93,94] and modelling of HIV in MSM in the UK[95].

M184V	0.2
K65R	0.2
Q151M	0.5
Thymidine analogue mutations (TAMS)	0.5
NNRTI mutations (K103N, G190A, Y181C)	1 - (0.20**res_trans_factor_nn*)
PI mutations	0.5
Dolutegravir mutations	1 - (0.20**res_trans_factor_ii*)

We consider uncertainty in the extent to which transmitted NNRTI and dolutegravir resistance mutations are effectively immediately lost (even from minority virus) by sampling from a distribution for parameter 1 - (0.20**res_trans_factor_nn*), informed by fitting of a model of HIV in MSM to UK data[95]. We also consider a similar parameter for dolutegravir.

Loss from majority virus of transmitted mutations

There is a probability per 3 months of loss of persistence of transmitted mutations from majority virus to minority virus (same for each mutation) *rate_loss_persistence*, again informed by fitting of a model of HIV in MSM to UK data[95].

Risk of clinical disease and death in HIV infected people

Occurrence of WHO 4 diseases

The rate of WHO 4 diseases according to CD4 count per 3 months is given below.

Table S9. Rate of WHO stage 4 disease according to CD4 count and viral load.

If cd4 ≥ 650	rate=0.002	if 500 ≤ cd4 < 650	rate=0.010
if 450 ≤ cd4 < 500	rate=0.013	if 400 ≤ cd4 < 450	rate=0.016
if 375 ≤ cd4 < 400	rate=0.020	if 350 ≤ cd4 < 375	rate=0.022
if 325 ≤ cd4 < 350	rate=0.025	if 300 ≤ cd4 < 325	rate=0.030
if 275 ≤ cd4 < 300	rate=0.037	if 250 ≤ cd4 < 275	rate=0.045
if 225 ≤ cd4 < 250	rate=0.055	if 200 ≤ cd4 < 225	rate=0.065
if 175 ≤ cd4 < 200	rate=0.080	if 150 ≤ cd4 < 175	rate=0.10
if 125 ≤ cd4 < 150	rate=0.13	if 100 ≤ cd4 < 125	rate=0.17
if 90 ≤ cd4 < 100	rate=0.20	if 80 ≤ cd4 < 90	rate=0.23
if 70 ≤ cd4 < 80	rate=0.28	if 60 ≤ cd4 < 70	rate=0.32
if 50 ≤ cd4 < 60	rate=0.40	if 40 ≤ cd4 < 50	rate=0.50
if 30 ≤ cd4 < 40	rate=0.80	if 20 ≤ cd4 < 30	rate=1.10
if 10 ≤ cd4 < 20	rate=1.80	if 0 ≤ cd4 < 10	rate=2.50
Independent effect of viral load			
if v < 3	rate = rate x 0.2		
if 3 <= v < 4	rate = rate x 0.3		
if 4 <= v < 4.5	rate = rate x 0.6		
if 4.5 <= v < 5	rate = rate x 0.9		
if 5 <= v < 5.5	rate = rate x 1.2		
if 5.5 <= v	rate = rate x 1.6		

This is informed by Phillips et al [96].

Independent effect of age

rate = rate x $(\text{age} / 38)^{1.2}$

Independent effect of PJP prophylaxis

If patient on PJP prophylaxis then this rate is multiplied by 0.8. If CD4 count is measured and current value < 350 /mm3 then patient assumed to have 80% chance of starting PJP prophylaxis after 1996. If patient has current WHO stage 3 or 4 condition they are assumed to have an 80% chance of starting PJP prophylaxis. If the CD4 count is measured then PJP prophylaxis assumed to stop if current value > 350/mm3. If the patient has been continuously on ART for 2 years with no WHO 3 or 4 condition in previous 6 months then it is assumed that PJP prophylaxis is stopped.

Independent effect of being on ART

For patients on a single drug regimen this risk is multiplied by 0.9, for patients on a two drug regimen it is multiplied by 0.85 and for patients on a 3 drug regimen it is multiplied by 0.6, to reflect that being on ART has a positive effect on risk of AIDS and death independent of latest CD4 count and viral load.

Occurrence of WHO 3 diseases

As for WHO 4 except risk is fold_incr_who3 (= 5) higher.

Risk of HIV-related death

As for WHO 4 except risk *fold_decr_hivdeath* - fold lower (= 0.25).

CD4-, viral load- age-specific death rate raised *incr_death_rate_tb*-fold (= 10) if current TB and *incr_death_rate_adc*-fold (= 10) if current WHO 4 disease. We assume 15% of HIV-related deaths (ie not including deaths that arise due to background mortality rates) are classified as non-HIV-related.

Pregnancy

We model pregnancy as occurring in the 3 month period in which the 9 month period is reached. The base rate of pregnancy relating to women aged 35-45 who had condomless sex in the relevant 3 month period is *prob_pregnancy_base* (see Table S10 below). This is multiplied by age specific probabilities, *fold_preg* to reflect lower likelihood of pregnancy in older women. The multiplicative factors for women ages 15-25, 25-35, 45-55 are 1.04, 1.03 and 0.3, respectively. For a women who had condomless sex with a short-term partner, the probability of pregnancy is multiplied by the factor *fold_tr_newp (=0.3)*, to take into account the lower number of sex acts per short term partner than per long term partner. Risk of mother to chold transmission is dependent on the viral load of the mother at birth: viral load > 100,000: 40% risk, 10,000 – 100,00: 20%, 1000 – 10,000: 10%, < 1000: 0.02%. Risk of NTD due to dolutegravir applies to women on dolutegravir in the relevant period of conception.

Table S10. Parameter distributions sampled for each model run together with the distribution for the 1000 selected setting-scenarios. Each model run creates one setting scenario. Setting scenarios were selected to be part of the 1000 if they fulfilled the following criteria in 2018: prevalence (age 15-49) > 2%, incidence (age 15-49) > 0.10 per 100 person years, percentage of women giving birth with HIV in whom there is transmission to the child between 3% and 0.25, percentage of people with HIV diagnosed between 48% and 97%, percentage of people diagnosed with HIV who are on ART > 68%, percentage of all people on ART who have viral load < 1000 copies/mL> 72% , percentage of all HIV positive people with viral load < 1000 copies/mL between 36% and 82%, percentage of people initiating ART who have resistance to efavirenz between 0.1% and 30%, percentage of women giving birth per year between 5% and 19%. The comparison of these setting scenarios with observed data is shown in Table 1 of the main paper.

Parameter name	Description	Distribution sampled / of 1000 setting scenarios (value; % with value)	Motivation for distribution
Parameters relating to sexual behaviour*			
swn	Value of multiplicative factor determining numbers of partners for those in highest new partner group (i.e. female sex workers)	4 20 / 10<br>8 20 / 18<br>12 20 / 21<br>16 20 / 26<br>20 20 / 26	This parameter helps to determine the extent to which the epidemic is driven by transactional sex, which is likely to vary in specific setting scenarios.
highsa	Value of and fold change in multiplicative factor determining numbers of partners for those in second highest new partner group	3 25 / 34<br>4 25 / 26<br>5 25 / 21<br>6 25 / 19	Range of values that was found, in certain (randomly selected) combination with other sexual behaviour parameter values to re-produce epidemics within the observed prevalence range. Note also that sexual behaviour tends to be under-reported, particularly in women, and higher levels of behaviour have to be assumed both to be consistent with levels of risk behaviour reported in men, and to generate an epidemic of the proportions observed (e.g.[97,98]).
sex_beh_trans_matrix	Matrix determining rate of transition between four levels of sexual behaviour. There are 15 versions for each of men and women.	1/15 probability for each transition matrix for men, same for women<br><br>% with each value:<br><br>women 1 9%, 2 7%, 3 6%, 4 8%, 5 9%, 6 3%, 7 11%, 8 9%, 9 6%, 10 8%, 11 6%, 12 2%, 13 8%, 14 6%, 15 3%<br><br>men 1 8%, 2 8%, 3 8%, 4 9%, 5 6%, 6 5%, 7 8%, 8 8%, 9 8%, 10 6%, 11 5%, 12 6%, 13 5%, 14 5%, 15 5%	Due to the fact that data on sexual behaviour are from self report, which is known to be highly unreliable, there is uncertainty over longitudinal patterns of sexual behaviour and the degree of skewness in the distribution of number of new partners we consider a range of possible matrices (15 for each gender = 225 possible combinations). Skewness is alo influenced by the *swn* parameter.
p_rred_p	Indicates the proportion of the population in whom the sexual risk behaviour is very low	0.1 20 / 25<br>0.2 20 / 22<br>0.3 20 / 17	In order to include a person-level effect on sexual behaviour this and the parameter below allow the population to be

		0.4 20 / 16<br>0.5 20 / 19	divided into three according to the lifelong tendency to have condomless sex.
p_hsb_p	Indicates the proportion of the population in whom the sexual risk behaviour has a tendency to be higher than average	0.02 20 / 3<br>0.05 20 / 10<br>0.1 20 / 23<br>0.15 20 / 31<br>0.2 20 / 33	As above
newp_factor	Overall average level of sexual risk behaviour. The correlation with the above parameters induced by the sampling of this parameter is to provide a focus on parameter space most likely to give low values of the overall fit. For example, if the sampling of *swn* and *highsa* give values at the high end of the distribution and sampling of *p_rred_p* produces a value at the low end then the model simulation run will produce an epidemic which is too large, unless there is some compensation when selecting the value of this parameter.	3 x (4.5/highsa)x(12/swn) x (p_rred_p/0.3) x (0.1/p_hsb_p) x exp(Normal(0, 0.5^2))<br><br>0.1 – 0.9 32<br>1 – 1.9 26%<br>2 – 2.9 13%<br>3 – 3.9 9%<br>4 – 6.9 9%<br>≥ 7 10%	See description of parameter
conc_ep	Parameter indicating the degree to which those with a long term condomless sex partner have a lower of higher probability of short term condomless sex partners than those without a long term condomless sex partner.	Lognormal(0,0.6)<br><br>0.1 – 0.9 57%<br>1.0 - 1.9 35%<br>≥ 2 8%	This is likely to vary across setting scenarios and we wished to consider across the range. Again, this distribution of values was found, in certain (randomly selected) combination with other sexual behaviour parameter values to re-produce epidemics within the observed prevalence range.
ych_risk_beh_newp	Degree of reduction in condomless sex with short term partners per year from 1995 – 2000	0.02 14 / 6<br>0.05 14 / 9<br>0.08 14 / 12<br>0.11 14 / 16<br>0.14 14 / 23<br>0.17 14 / 32<br>0.20 16 / 2	In order to explain the decrease in incidence and prevalence of HIV in southern Africa in the late 1990s it is necessary to assume there was a reduction in condomless sex, which is supported by data in Zimbabwe, for example [99,100]
ych_risk_beh_ep	Degree of reduction in condomless sex per year with long term partners from 1995-2000	0 20 / 21<br>0.02 20 / 22<br>0.04 20 / 20<br>0.06 20 / 18<br>0.08 20 / 19	As above
ch_risk_diag_newp	Degree of reduction (fold change) in condomless sex with short term partners in a person diagnosed with HIV	0.7 25 / 25<br>0.8 25 / 22<br>0.9 25 / 25<br>1 20 / 28	Informed by [101]
ch_risk_diag	Degree of reduction in condomless sex with long term partner in a person diagnosed with HIV	0.7 30 / 24<br>0.8 25 / 26<br>0.9 25 / 25<br>1 20 / 20	Informed by [101]
ych2_risk_beh_newp	Degree of change in condomless sex with short term partners per year from 2010 – 2015	-0.04 5 / 7<br>-0.02 5 / 5<br>0 80 / 80	It is uncertain whether there have been recent changes in condomless sex, hence a neutral distribution was used.

		0.02 5 / 4<br>0.04 5 / 4	
**Parameters relating to transmission***			
fold_change_w	The fold difference in female to males transmission rate compared with male to female, for a given viral load.	1 5 / 6<br>1.25 5 / 6<br>1.5 90 / 88	Informed by the higher incidence and prevalence in women in younger age groups and some direct evidence.
res_trans_factor_nn	Parameter determining the probability that if NNRTI resistance mutation present in source partner that this is not present/detectable in virus new host	90% chance of transmision 20 / 20<br>86% chance of transmission 20 / 19<br>84% chance of transmision 20 / 22<br>82% chance of transmission 20 / 21<br>80% chance of transmission 20 / 18	Informed by the values needed to lead to the range of transmitted NNRTI resistance observed [102-104]
res_trans_factor_ii	Parameter determining the probability that if integrase inhibitor resistance mutation present in source partner that this is not present/detectable in virus new host	80% chance of transmision 80 / 81<br>60% chance of transmission 20 / 19	Little data available to inform this.
**Parameters relating to HIV testing***			
an_lin_incr_test	Parameter determining the rate of increase in HIV testing (any testing outside ANC)	0.0001 17 / 14<br>0.0005 17 / 16<br>0.001 17 / 15<br>0.005 17 / 20<br>0.01 17 / 18<br>0.015 15 / 16	Range and pattern required to re-produce the observed range in proportion of HIV positive people diagnosed (see Table 2 of main paper).
*date_test_rate_plateau_*	Year in which the rate of HIV testing plateaus.	2011.5 25 / 26<br>2013.5 25 / 23<br>2015.5 25 / 27<br>2017.5 25 / 25	Some countries have increased testing rates markedly and these have plateaued at different levels in different settings (e.g Government of Malawi Ministry of Health Quarterly Reports).
rate_testanc_inc	Rate of increase in testing in ANC clinics	0 20 / 17<br>0.01 20 / 20<br>0.03 20 / 21<br>0.05 20 / 21<br>0.1 20 / 20	Government of Malawi Ministry of Health Quarterly Reports. Again distribution is intended to reflect variation across setting scenarios.
*incr_test_rate_sympt_*	The rate of increase over time in the probability of a person with a WHO stage 3 or 4 disease is tested for HIV.	1.02 20 / 18<br>1.05 20 / 21<br>1.10 20 / 21<br>1.20 20 / 20<br>20 20 / 20	Little direct data on this parameter and wide range taken to reflect uncertainty and variation across settings.
**Parameters relating to pre-ART care and progression of HIV***			
fx	Multiplicative factor to alter the average rate of CD4 count decline in natural HIV progression (which thus alters the incubation period distribution).	0.7 20 / 20<br>0.8 20 / 20<br>0.9 20 / 20<br>1 20 / 21	Derived based on consideration of evidence from natural history studies [105-113].

		1.1	20 / 19	
prob_loss_at_diag	Probability that a person is immediately lost after initial HIV diagnosis.	0.02<br>0.05<br>0.15<br>0.25<br>0.35	25 / 23<br>15 / 17<br>30 / 32<br>25 / 23<br>5 / 5	Rosen at al[114]
rate_lost	For people under care yet to start ART or previously have taken ART, the rate of being lost to care per 3 mths.	0.05<br>0.1<br>0.15<br>0.3<br>0.5	20 / 20<br>40 / 41<br>20 / 20<br>10 / 9<br>10 / 9	Uncertain and will vary by setting. Distribution chosen to reflect this. This is one of the parameters influencing the proportion of diagnosed people on ART.
rate_return	Probability of return to care for a person who has been diagnosed with HIV (and may have started ART) but is now lost and not on ART, without current WHO stage 3 or 4 disease, per 3 months.	0.05<br>0.1<br>0.15<br>0.2<br>0.5	15 / 14<br>10 / 10<br>25 / 23<br>25 / 25<br>25 / 29	As above
prob_return_adc	Probability of return to care for a person who has been diagnosed with HIV (and may have started ART) but is now lost and not on ART and has a WHO stage 4 condition. This is a probability that operates just for the 3 month period that the events occurs.	0.2<br>0.4<br>0.6<br>0.8	5 / 6<br>15 / 14<br>30 / 28<br>50 / 52	As above
rate_loss_persistence	Rate of loss from majority virus of transmitted resistance mutations (per 3 months)	0<br>0.005<br>0.01<br>0.015<br>0.02	10 / 11<br>10 / 9<br>10 / 11<br>40 / 39<br>30 / 29	[91, 115-116]
**Parameters relating to people on ART**				
adh_pattern	Population adherence profile; described in terms of the proportion having a given average adherence and period-to-period variability in adherence. Note that adherence is additonally affected by age and gender as described on page 7.	A<br>B<br>C<br>D<br>E<br>F<br>G<br>H<br>I	3 / 3<br>3 / 4<br>6 / 6<br>4 / 4<br>9 / 9<br>10 / 12<br>15 / 21<br>20 / 25<br>30 / 17	Reflection of wide range of adherence profiles in different settings, informed by differences in proportions of people on ART with viral load suppression.
pr_art_init	Probability of ART initiation per 3 months in a person in care who is eligible according to current criteria.	0.4<br>0.5<br>0.6<br>0.7	25 / 24<br>25 / 27<br>25 / 23<br>25 / 26	These parameters contribute to determine the proportion of HIV diagnosed people who are on ART. The distributions are chosen such that combinations of these parameters lead to observed proportions of HIV diagnosed people on ART (e.g. Population Health Impact Surveys [29])
prob_lost_art	For a person who interrupts / stops ART the probability that they are simultaneously lost from care.	0.5<br>0.6	20 / 21<br>20 / 20	[43, 44, 117]

		0.7<br>0.8<br>0.9	20 / 18<br>20 / 22<br>20 / 20	
rate_restart	Rate of restart of ART for people who previously have been on ART and have returned to care, per 3 months.	0.2<br>0.4<br>0.6<br>0.8	25 / 24<br>25 / 26<br>25 / 28<br>25 / 22	[43] Assumed to be high, given the person has returned to care. Most people who are regularly seen in clinics who have previously started ART are on ART.
rate_int_choice	Rate of interruption / stopping of ART per 3 months. Also influenced by current drug toxicity and underlying tendency to adhere.	0.001<br>0.005<br>0.01<br>0.015<br>0.02<br>0.025	20 / 21<br>10 / 11<br>10 / 10<br>20 / 20<br>20 / 21<br>20 / 17	[43, 44, 117]
incr_rate_int_low_adh	Parameter indicating the extent to which people with a long term average adherence in the lowest group have a multiplicatively increased risk of ART interruption.	1<br>2<br>5	50 / 49<br>25 / 25<br>25 / 26	[118]
pr_switch_line	Probability of switch to second line per 3 months in a person who has fulfilled the failure criteria for first line failure.	0.20<br>0.50	50 / 50<br>20 / 50	[2,3]. In several settings, including Zimbabwe, the proportion of people who have started second line ART is consistent with a value for *pr_switch*_line of below 0.1 (e.g. Lesotho, Malawi) (Government of Malawi MoH Quarterly Reports).
clinic_not_aw_int_frac	If a person interrupts ART, the probability that this is not disclosed to the clinic and they are classified as being on ART	0.1<br>0.3<br>0.5<br>0.7<br>0.9	20 / 22<br>20 / 19<br>20 / 19<br>20 / 20<br>20 / 20	Uncertain and will vary by setting, hence a broad distribution.
fold_change_mut_risk	Fold difference in rate of accumulation of mutations (for all drugs) compared with base case.	0.5<br>1	36 / 54<br>80 / 46	To consider that the rate of resistance mutation acquisition is higher or lower than the rate assumed. This relates to all resistance mutations.
*rate_res_ten_*	Parameter reflecting the rate of acquisition of tenofovir resistance. The value of 0.1 was derived based on European cohort data and the value of 0.3 reflects the potentially higher value for subtype C in southern Africa.	0.1<br>0.3	10 / 11<br>90 / 89	[119]
incr_rate_int_low_adh	Effect of current low adherence on risk of treatment interruption / discontinuation.	1<br>2<br>5	50 / 49<br>25 / 25<br>25 / 26	Low adherence predicts interruption of ART (unpublished data).
poorer_cd4_rise_on_fail_nn_ii	This indicates whether the poorer CD4 rise on failing NNRTI based regimens (compared with PI) also holds for dolutegravir-based regimens.	no<br>yes	50 / 49<br>50 / 51	The 50% with yes may be over-pessimistic regarding effects of dolutegravir as CD4 count responses are superior compared with efavirenz.
adh_effect_of_meas_alert	The effect of having a viral load measured > 1000 copies/mL on adherence, due to the enhanced adherence intervention.	0.35<br>0.7<br>0.9	15 / 14<br>70 / 69<br>15 / 18	Uncertainty over the effect size.

prob_vl_meas_done	Probability of a viral loead measure being done. This probability operates for each time a viral load is due to be tested.	0.00 25 / 36<br>0.10 25 / 20<br>0.25 25 / 21<br>0.85 25 / 23	Variation in viral load implementation in different settings. Note that in half of settings with value 0 there is CD4 count monitoring of people on ART in place (see below).
cd4_monitoring	If *prob_vl_meas_done* = 0 then is CD4 count monitoring in place ?	no 50 / 51<br>yes 50 / 49	Uncertain to what extent countries or settings where viral load testing is not being implemented are using CD4 count monitoring.
zero_3tc_activity_m184	activity of 3TC in presence of M184V mutation	0.25 activity 80 / 79<br>0.00 activity 20 / 21	To consider alternative assumptions; distribution broadly reflects the uncertainty.
zero_ten_activity_k65	activity of 3TC in presence of K65R mutation	0.25 activity 80 / 79<br>0.00 activity 20 / 21	To consider alternative assumptions; distribution broadly reflects the uncertainty.
higher_rate_res_dol	Whether there is a higher rate of resistance to dolutegravir than the base assumption (i.e. 4 times lower than efavirenz compared with 13 times lower in base case).	no 80 / 80<br>yes 20 / 20	To consider alternative assumptions; distribution broadly reflects the uncertainty.
*dol_higher_potency_*	Potency (relative to efavirenz and other drugs apart from boosted PI)	1.5 100 / 100	Previously we considered higher values for potency of dolutegravir compared with eafvirenz [120-140]. In the light of results from NAMSAL and ADVANCE we modified so that both drugs have a potency 1.5. [141-142]
*rel_dol_tox_*	Relative rate of neurologic toxicity (sleep disturbance for dolutegravir and dizziness and vivid dreams for efavirenz)	0.5 fold that of efavirenz 80 / 81<br>Equal to efavirenz 20 / 19	While evidence suggests neurologic toxicity is higher with efavirenz, there is uncertainty over size of effect of insomnia with dolutegravir so we consider the possibility that the overall neurologic toxicity of the two drugs could be equal.
*prop_bmi_ge23_*	Proportion of people initiating dolutegravir who have BMI $\geq$ 23	50% 50 / 49<br>75% 50 / 51	uncertainty over the proportion of the population starting dolutegarvir who have BMI > 23 and hence a possible negative effect of weight gain on dolutegravir. [143-144]
*incr_mort_risk_dol_weightg_*	Rate ratio for mortality in people on dolutegravir who had BMI $\geq$ 23 at start, due to dolutegravir induced weight gain.	1.00 1 / 1<br>1.03 16 / 13<br>1.05 17 / 18<br>1.07 17 / 14<br>1.10 17 / 17<br>1.15 17 / 19<br>1.25 15 / 17	Wide distribution within plausible bounds reflecting uncertainty [145-148].
nnrti_res_no_effect	Effect of NNRTI resistance mutations on activity of efavirenz (base case: K103N 0 activity, G190A 0.25 activity, Y181C 0.25 activity)	base case 75 / 76<br>each mutation leaves 0.25 activity 20 / 20<br>each mutation leaves 0.50 activity 5 / 4	Some uncertainty over this. With this distribution the average odds ratio for VL > 1000 at 1 year from start of ART associated with pre-treatment NNRTI drug resistance = 3.3 (compared with 3.9 in a recent meta-analysis [149.]
tox_weightg_dol	Whether weight gain is treated as a toxicity that has an associated increased risk of ART interruption	No 50% 50 / 49<br>Yes 50% 50 / 51	Weight gain does not seem to be mentioned as troublesome to people on dolutegravir but this could change with time.
double_rate_gas_tox_taz	Parameter related to the rate of gastrointestinal toxity relating to atazanavir. Whether base rate is doubled or not.	No 50 / 51<br>Yes 50 / 49	Uncertainty over gastrointestinal toxity relating to atazanavir – consider possibility that this has been underestimated.

rr_int_tox	Increased rate of ART interruption according to presence of a drug toxicity.	2 fold 50 / 49<br>10 fold 50 / 51	Consider possibiity that ART interruption is substantially more highly related to drug toxicity than base case
**Parameter relating to pregnancy**			
prob_pregnancy_base	Parameter determining base rate of pregnancy for women having condomless sex(to which there is an effect of age)	Uniform (7%, 22%)	Variability between settings.
rate_birth_with_infected_child	Parameter determining the risk of MTCT, for a given level of mother viral load.	0.3 5 / 5<br>0.4 25 / 25<br>0.5 60 / 61<br>0.6 10 / 10	To produce plausible variation in the MTCT rate.
*oth_dol_adv_birth_e_risk_*	Risk of dolutegravir-induced adverse birth event, due to dolutegravir-induced weight gain	0.05% 20 / 20<br>0.15% 40 / 38<br>0.20% 20 / 21<br>0.30% 20 / 21	Wide distribution within plausible bounds reflecting uncertainty. [150]

Model runs are not accepted as "setting scenarios" if HIV prevalence in 2017 is < 5% or HIV incidence is > 1.6 per 100 person years.
* Further details of modelling of demographics, sexual behaviour, HIV transmission and HIV testing and associated parameters are explained in detail in a supplement to a recent paper[1] and can be found here: https://www.thelancet.com/cms/10.1016/S2352-3018(17)30190-X/attachment/02742987-df48-4372-8e4a-43888c2ec1e8/mmc1.pdf

Table S11. Disability weights

Values are 1 in each three month period except for the following:

Condition in current 3 month period	Disability weight for current 3 month period	Source
Any drug toxicity in current 3-month period	0.95	[151]
Any WHO stage 3 condition (except TB) in current 3-month period	0.78	[151]
TB in current 3-month period	0.60	[151]
Any WHO stage 4 condition in current 3-month period	0.46	[151]

Costs

Table S12. Unit Costs.

Item	Unit Cost	Source / explanation
Drug costs per year:<br>TLE<br>TLD<br>ZL-PI (PI atazanavir)<br>ZLD	$90 ($75 without supply chain costs<br>$90 ($75 without supply chain costs)<br>$318 ($265 without supply chain costs)<br>$126 ($105 without supply chain costs)	[152]
Cost of treatment of a WHO stage 4 condition over 3 months (cost is incurred for 3 months)<br>Cost of treatment of a WHO stage 3 condition over 3 months (cost is incurred for 3 months)<br>Cost of treatment of TB per 3 months (cost is incurred for 6 months)<br>Cotrimoxazole annual cost	$200<br>$20<br>$50<br>$5	Specific data not available on average unit costs of treating WHO stage 3 and 4 conditions and per clinic visit costs - costs used are informed by evidence synthesis from studies that cost according to current CD4 count of those in pre-ART care, cost of ART initiation, which also include costs of CD4 tests [153]
CD4 count measurement	$10	[154,155]
Viral load measurement:	$22	Human resource costs $3, sample collection consumables $2, relaying of results $2 (this costing information was provided by Medecin Sans Frontiers (MSF) (including equipment and other costs such as consumables, maintenance and shipping) $15. Updates are consistent with this cost [136, 157].
Non-ART programme costs per year, $40 per year if on tiered care due to viral load < 1000	$80	[158-160] Bill and Melinda Gates Foundation tiered care meeting report (the per client cost of running the Khayelitsha adherence clubs was $58 per client per year compared to standard clinic care of $108 per client per year. At the Infectious Disease Institute in Kampala, the annual costs per client for physician, nurse, and pharmacy only visits were $60, $45, and $19, respectively)

Cost of the targeted adherence counselling intervention triggered by a viral load > 1000 copies/mL	$10	Assumption
HIV test (including personnel costs)	$3.70	Personal communication. CHAI.
Annual cost of treatment for a child born with HIV	$160	This cost was estimated based on a drug cost of $75 per year, a one-off cost of early infant diagnosis of $22, cost of viral load testing of $22 per year, costs of clinic visits of $40 or $80 per year (depending on whether viral load is suppressed), assuming 50% of children wll have viral suppression. This is likely to be a lower limit cost per year.

References

1. Phillips AN, Cambiano V, Nakagawa F, Revill P, Jordan MR, Hallett TB et al. Cost-effectiveness of public-health policy options in the presence of pretreatment NNRTI drug resistance in sub-Saharan Africa: a modelling study Lancet HIV 2018; 5: E146-E154 DOI: 10.1016/S2352-3018(17)30190-X
2. Fox MP et al. Rates and Predictors of Failure of First-line Antiretroviral Therapy and Switch to Second-line ART in South Africa JAIDS 2012; 60:428–437
3. Johnston, V., Fielding, K.L., Charalambous, S., Churchyard, G., Phillips, A., & Grant, A.D. 2012. Outcomes following virological failure and predictors of switching to second-line antiretroviral therapy in a South African treatment program. J.Acquir.Immune.Defic.Syndr., 61, (3) 370-380 available from: PM:22820803
4. Fox Z, Phillips AN, Cohen C, et al. Viral resuppression and detection of drug resistance following interruption of a suppressive non-nucleoside reverse transcriptase inhibitor-based regimen. AIDS 2008; 22:2279-2289.
5. Bangsberg DR, Moss AR, Deeks SG et al. Paradoxes of adherence and drug resistance to HIV antiretroviral therapy. J Antimicrob Chem 2004; 53 (5): 696-699.
6. Bangsberg, D.R., Acosta, E.P., Gupta, R., Guzman, D., Riley, E.D., Harrigan, P.R., Parkin, N., & Deeks, S.G. 2006. Adherence-resistance relationships for protease and non-nucleoside reverse transcriptase inhibitors explained by virological fitness. *AIDS*, 20, (2) 223-231 available from: PM:16511415
7. Bangsberg, D.R. 2006. Less than 95% adherence to nonnucleoside reverse-transcriptase inhibitor therapy can lead to viral suppression. *Clin.Infect.Dis.*, 43, (7) 939-941 available from: PM:16941380
8. Hamers, R.L., Wallis, C.L., Kityo, C., Siwale, M., Mandaliya, K., Conradie, F., Botes, M.E., Wellington, M., Osibogun, A., Sigaloff, K.C., Nankya, I., Schuurman, R., Wit, F.W., Stevens, W.S., van, V.M., & de Wit, T.F. 2011. HIV-1 drug resistance in antiretroviral-naive individuals in sub-Saharan Africa after rollout of antiretroviral therapy: a multicentre observational study. *Lancet Infect.Dis.*, 11, (10) 750-759 available from: PM:21802367
9. Hassan, A.S., Nabwera, H.M., Mwaringa, S.M., Obonyo, C.A., Sanders, E.J., Rinke de Wit, T.F., Cane, P.A., & Berkley, J.A. 2014. HIV-1 virologic failure and acquired drug resistance among first-line antiretroviral experienced adults at a rural HIV clinic in coastal Kenya: a cross-sectional study. *AIDS Res.Ther.*, 11, (1) 9 available from: PM:24456757
10. Hoffmann CJ, Charalambous S, Sim J, et al. Viremia, Resuppression, and Time to Resistance in Human Immunodeficiency Virus (HIV) Subtype C during First-Line Antiretroviral Therapy in South Africa. Clin Infect Dis 2009; 49:1928–35.
11. Hoffmann, C.J., Charalambous, S., Grant, A.D., Morris, L., Churchyard, G.J., & Chaisson, R.E. 2014. Durable HIV RNA resuppression after virologic failure while remaining on a first-line regimen: a cohort study. *Trop.Med.Int.Health*, 19, (2) 236-239 available from: PM:24588012
12. Kobin, A.B. & Sheth, N.U. 2011. Levels of adherence required for virologic suppression among newer antiretroviral medications. *Ann.Pharmacother.*, 45, (3) 372-379 available from: PM:21386024
13. Li, J.Z., Gallien, S., Ribaudo, H., Heisey, A., Bangsberg, D.R., & Kuritzkes, D.R. 2014. Incomplete adherence to antiretroviral therapy is associated with higher levels of residual HIV-1 viremia. *AIDS*, 28, (2) 181-186 available from: PM:24361679
14. Mackie, N.E., Phillips, A.N., Kaye, S., Booth, C., & Geretti, A.M. 2010. Antiretroviral drug resistance in HIV-1-infected patients with low-level viremia. J.Infect.Dis., 201, (9) 1303-1307 available from: PM:20350161
15. Rosenblum, M., Deeks, S.G., van der Laan, M., & Bangsberg, D.R. 2009. The risk of virologic failure decreases with duration of HIV suppression, at greater than 50% adherence to antiretroviral therapy. PLoS.One., 4, (9) e7196 available from: PM:19787058
16. Tran, D.A., Wilson, D.P., Shakeshaft, A., Ngo, A.D., Doran, C., & Zhang, L. 2014. Determinants of virological failure after 1 year's antiretroviral therapy in Vietnamese people with HIV: findings from a retrospective cohort of 13 outpatient clinics in six provinces. *Sex Transm.Infect.* available from: PM:24619575

17. Usitalo, A., Leister, E., Tassiopoulos, K., Allison, S., Malee, K., Paul, M.E., Smith, R., Van Dyke, R.B., Seage, G.R., III, & Mellins, C.A. 2014. Relationship between viral load and self-report measures of medication adherence among youth with perinatal HIV infection. *AIDS Care*, 26, (1) 107-115 available from: PM:23800360
18. von Wyl., V, Klimkait, T., Yerly, S., Nicca, D., Furrer, H., Cavassini, M., Calmy, A., Bernasconi, E., Boni, J., Aubert, V., Gunthard, H.F., Bucher, H.C., & Glass, T.R. 2013. Adherence as a predictor of the development of class-specific resistance mutations: the Swiss HIV Cohort Study. *PLoS.One.*, 8, (10) e77691 available from: PM:24147057
19. Johannessen, A., Naman, E., Kivuyo, S.L., Kasubi, M.J., Holberg-Petersen, M., Matee, M.I., Gundersen, S.G., & Bruun, J.N. 2009. Virological efficacy and emergence of drug resistance in adults on antiretroviral treatment in rural Tanzania. *BMC.Infect.Dis.*, 9, 108 available from: PM:19583845
20. Muyingo, S.K., Walker, A.S., Reid, A., Munderi, P., Gibb, D.M., Ssali, F., Levin, J., Katabira, E., Gilks, C., & Todd, J. 2008. Patterns of individual and population-level adherence to antiretroviral therapy and risk factors for poor adherence in the first year of the DART trial in Uganda and Zimbabwe. *J.Acquir.Immune.Defic.Syndr.*, 48, (4) 468-475 available from: PM:18614918
21. Walmsley SL, Antela A, Clumeck N, Duiculescu D, Eberhard A, Gutierrez F, et al. Dolutegravir plus abacavir-lamivudine for the treatment of HIV-1 infection. N Engl J Med. 2013; 369(19):1807–18. doi: 10.1056/NEJMoa1215541 PMID: 24195548
22. Walmsley S, Baumgarten A, Berenguer J, Felizarta F, Florence E, Khuong-Josses MA, et al. Brief Report: Dolutegravir Plus Abacavir/lamivudine for the Treatment of HIV-1 Infection in Antiretroviral Therapy-Naive Patients: Week 96 and Week 144 Results From the SINGLE Randomized Clinical Trial. J Acquir Immune Defic Syndr. 2015;70(5):515–9. doi: 10.1097/QAI.0000000000000790. pmid:26262777
23. O'Connor JL, Gardner EM, Esser S, Mannheimer SB, Lifson AR, Telzak E, et al. A simple self-reported adherence tool as a predictor of viral rebound in people with viral suppression on antiretroviral therapy. HIV Medicine (2016), 17, 124—132.
24. Filimão DBC, Moon TD, Senise JF, Diaz RS, Sidat M, Castelo A (2019) Individual factors associated with time to non-adherence to ART pick-up within HIV care and treatment services in three health facilities of Zambe´zia Province, Mozambique. PLoS ONE 14(3): e0213804. https://doi.org/10.1371/journal.pone.0213804
25. Haberer JE, Bosco M, Bwana M, Orrell C, Asiimwe S, Amanyire G, Musinguzi N et al. ART adherence and viral suppression are high among most non-pregnant individuals with early-stage, asymptomatic HIV infection: an observational study from Uganda and South Africa JIAS 2019, 22:e25232.
26. Liegeois F, Eymard-Duvernay S, Boyer S, Maradan G, Kouanfack C, Domyeum J, et al. Heterogeneity of virological suppression in the national antiretroviral programme of Cameroon (ANRS 12288 EVOLCAM) HIV Medicine 2019; 20, 38—46
27. Jiamsakul A, Kariminia A, Althoff KN, Cesar C, Cortes CP, Davies M-A, et al. HIV Viral Load Suppression in Adults and Children Receiving Antiretroviral Therapy—Results From the IeDEA Collaboration. J Acquir Immune Defic Syndr 2017;76:319–329
28. Malawi Ministry of Health. Quarterly report 2018 3rd quarter
29. Population Health Impact Surveys. https://phia.icap.columbia.edu/
30. Chi, B.H., Cantrell, R.A., Zulu, I., Mulenga, L.B., Levy, J.W., Tambatamba, B.C., Reid, S., Mwango, A., Mwinga, A., Bulterys, M., Saag, M.S., & Stringer, J.S. 2009. Adherence to first-line antiretroviral therapy affects non-virologic outcomes among patients on treatment for more than 12 months in Lusaka, Zambia. Int.J.Epidemiol., 38, (3) 746-756 available from: PM:19223334
31. WHO HIV Drug Resistance Surveillance Report 2012 http://apps.who.int/iris/bitstream/handle/10665/75183/9789241503938_eng.pdf;jsessionid=B20E426C5A757C5F3DC01FA62A9F4F06?sequence=1
32. Cozzi-Lepri, A., UK HIV Drug Resistance, & UK CHIC 2010. Long-term probability of detecting drug-resistant HIV in treatment-naive patients initiating combination antiretroviral therapy. Clin.Infect.Dis., 50, (9) 1275-1285 available from: PM:20353366

33. Cheeseman, S.H., Hattox, S.E., McLaughlin, M.M., Koup, R.A., Andrews, C., Bova, C.A., Pav, J.W., Roy, T., Sullivan, J.L., & Keirns, J.J. 1993. Pharmacokinetics of nevirapine: initial single-rising-dose study in humans. Antimicrob.Agents Chemother., 37, (2) 178-182 available from: PM:8452345
34. Gardner, E.M., Burman, W.J., Steiner, J.F., Anderson, P.L., & Bangsberg, D.R. 2009. Antiretroviral medication adherence and the development of class-specific antiretroviral resistance. AIDS, 23, (9) 1035-1046 available from: PM:19381075
35. Gross, R., Bilker, W.B., Wang, H., & Chapman, J. 2008. How long is the window of opportunity between adherence failure and virologic failure on efavirenz-based HAART? HIV.Clin.Trials, 9, (3) 202-206 available from: PM:18547907
36. Meresse, M., March, L., Kouanfack, C., Bonono, R.C., Boyer, S., Laborde-Balen, G., Aghokeng, A., Suzan-Monti, M., Delaporte, E., Spire, B., Carrieri, M.P., & Laurent, C. 2014. Patterns of adherence to antiretroviral therapy and HIV drug resistance over time in the Stratall ANRS 12110/ESTHER trial in Cameroon. HIV.Med. available from: PM:24589279
37. Parienti, J.J., Massari, V., Reliquet, V., Chaillot, F., Le, M.G., Arvieux, C., Vabret, A., & Verdon, R. 2007. Effect of twice-daily nevirapine on adherence in HIV-1-infected patients: a randomized controlled study. AIDS, 21, (16) 2217-2222 available from: PM:18090049
38. Hill, A., McBride, A., Sawyer, A.W., Clumeck, N., & Gupta, R.K. 2013. Resistance at virological failure using boosted protease inhibitors versus nonnucleoside reverse transcriptase inhibitors as first-line antiretroviral therapy--implications for sustained efficacy of ART in resource-limited settings. J.Infect.Dis., 207 Suppl 2, S78-S84 available from: PM:23687293
39. Orrell, C., Harling, G., Lawn, S.D., Kaplan, R., McNally, M., Bekker, L.G., & Wood, R. 2007. Conservation of first-line antiretroviral treatment regimen where therapeutic options are limited. Antivir.Ther., 12, (1) 83-88 available from: PM:17503751
40. Rutstein SE, Hosseinipour MC, Kamwendo D, Soko A, Mkandawire M, Biddle AK, et al. (2015) Dried Blood Spots for Viral Load Monitoring in Malawi: Feasible and Effective. PLoS ONE 10(4): e0124748. doi:10.1371/journal.pone.0124748
41. Bonner, K., Mezochow, A., Roberts, T., Ford, N., & Cohn, J. 2013. Viral load monitoring as a tool to reinforce adherence: a systematic review. J.Acquir.Immune.Defic.Syndr., 64, (1) 74-78 available from: PM:23774877
42. Bärnighausen T, Chaiyachati K, Chimbindi N, et al. Interventions to increase antiretroviral adherence in sub-Saharan Africa: a systematic review of evaluation studies. Lancet Infect Dis 2011; 11: 942–51.
43. Kranzer, K. & Ford, N. Unstructured treatment interruption of antiretroviral therapy in clinical practice: a systematic review. Trop.Med.Int.Health, 2011; 16, (10) 1297-1313 available from: PM:21718394
44. Kranzer, K., Lewis, J.J., Ford, N., Zeinecker, J., Orrell, C., Lawn, S.D., Bekker, L.G., & Wood, R. 2010. Treatment interruption in a primary care antiretroviral therapy program in South Africa: cohort analysis of trends and risk factors. J.Acquir.Immune.Defic.Syndr., 55, (3) e17-e23 available from: PM:20827216
45. Tassie, J.M., Baijal, P., Vitoria, M.A., Alisalad, A., Crowley, S.P., & Souteyrand, Y. 2010. Trends in retention on antiretroviral therapy in national programs in low-income and middle-income countries. J.Acquir.Immune.Defic.Syndr., 54, (4) 437-441 available from: PM:20351559
46. Wandeler, G., Keiser, O., Pfeiffer, K., Pestilli, S., Fritz, C., Labhardt, N.D., Mbofana, F., Mudyiradima, R., Emmel, J., Egger, M., & Ehmer, J. 2012. Outcomes of antiretroviral treatment programs in rural Southern Africa. J.Acquir.Immune.Defic.Syndr., 59, (2) e9-16 available from: PM:22067665
47. Wallis, C.L., Mellors, J.W., Venter, W.D., Sanne, I., & Stevens, W. 2010. Varied patterns of HIV-1 drug resistance on failing first-line antiretroviral therapy in South Africa. J.Acquir.Immune.Defic.Syndr., 53, (4) 480-484 available from: PM:19801944
48. McMahon, J.H., Elliott, J.H., Bertagnolio, S., Kubiak, R., & Jordan, M.R. 2013. Viral suppression after 12 months of antiretroviral therapy in low- and middle-income countries: a systematic review. Bull.World Health Organ, 91, (5) 377-385E available from: PM:23678201 Charurat, M., Oyegunle, M., Benjamin, R., Habib, A., Eze, E., Ele, P., Ibanga, I., Ajayi, S., Eng, M., Mondal, P., Gebi, U., Iwu, E., Etiebet, M.A., Abimiku, A., Dakum, P., Farley, J., & Blattner, W. 2010. Patient retention and adherence to antiretrovirals

in a large antiretroviral therapy program in Nigeria: a longitudinal analysis for risk factors. PLoS.One., 5, (5) e10584 available from: PM:20485670

49. DeGruttola, V., Dix, L., D'Aquila, R., Holder, D., Phillips, A., Ait-Khaled, M., Baxter, J., Clevenbergh, P., Hammer, S., Harrigan, R., Katzenstein, D., Lanier, R., Miller, M., Para, M., Yerly, S., Zolopa, A., Murray, J., Patick, A., Miller, V., Castillo, S., Pedneault, L., & Mellors, J. 2000. The relation between baseline HIV drug resistance and response to antiretroviral therapy: re-analysis of retrospective and prospective studies using a standardized data analysis plan. Antivir.Ther., 5, (1) 41-48 available from: PM:10846592
50. Grinsztejn, B., Nguyen, B.Y., Katlama, C., Gatell, J.M., Lazzarin, A., Vittecoq, D., Gonzalez, C.J., Chen, J., Harvey, C.M., & Isaacs, R.D. 2007. Safety and efficacy of the HIV-1 integrase inhibitor raltegravir (MK-0518) in treatment-experienced patients with multidrug-resistant virus: a phase II randomised controlled trial. Lancet, 369, (9569) 1261-1269 available from: PM:17434401
51. Rosenbloom, D.I., Hill, A.L., Rabi, S.A., Siliciano, R.F., & Nowak, M.A. 2012. Antiretroviral dynamics determines HIV evolution and predicts therapy outcome. Nat.Med., 18, (9) 1378-1385 available from: PM:22941277
52. Genberg, B.L., Wilson, I.B., Bangsberg, D.R., Arnsten, J., Goggin, K., Remien, R.H., Simoni, J., Gross, R., Reynolds, N., Rosen, M., & Liu, H. 2012. Patterns of antiretroviral therapy adherence and impact on HIV RNA among patients in North America. AIDS, 26, (11) 1415-1423 available from: PM:22767342
53. Cambiano, V., Lampe, F.C., Rodger, A.J., Smith, C.J., Geretti, A.M., Lodwick, R.K., Holloway, J., Johnson, M., & Phillips, A.N. 2010b. Use of a prescription-based measure of antiretroviral therapy adherence to predict viral rebound in HIV-infected individuals with viral suppression. HIV.Med., 11, (3) 216-224 available from: PM:20002781
54. Arnsten, J.H., Demas, P.A., Farzadegan, H., Grant, R.W., Gourevitch, M.N., Chang, C.J., Buono, D., Eckholdt, H., Howard, A.A., & Schoenbaum, E.E. 2001. Antiretroviral therapy adherence and viral suppression in HIV-infected drug users: comparison of self-report and electronic monitoring. Clin.Infect.Dis., 33, (8) 1417-1423 available from: PM:11550118
55. Montaner, J.S., Reiss, P., Cooper, D., Vella, S., Harris, M., Conway, B., Wainberg, M.A., Smith, D., Robinson, P., Hall, D., Myers, M., & Lange, J.M. A randomized, double-blind trial comparing combinations of nevirapine, didanosine, and zidovudine for HIV-infected patients: the INCAS Trial. Italy, The Netherlands, Canada and Australia Study. JAMA 1998, 279, (12) 930-937 available from: PM:9544767
56. Eron, J.J., Benoit, S.L., Jemsek, J., MacArthur, R.D., Santana, J., Quinn, J.B., Kuritzkes, D.R., Fallon, M.A., & Rubin, M. 1995. Treatment with lamivudine, zidovudine, or both in HIV-positive patients with 200 to 500 CD4+ cells per cubic millimeter. North American HIV Working Party. N.Engl.J.Med., 333, (25) 1662-1669 available from: PM:7477218
57. Havlir, D., McLaughlin, M.M., & Richman, D.D. 1995. A pilot study to evaluate the development of resistance to nevirapine in asymptomatic human immunodeficiency virus-infected patients with CD4 cell counts of > 500/mm3: AIDS Clinical Trials Group Protocol 208. J.Infect.Dis., 172, (5) 1379-1383 available from: PM:7594683
58. Kuritzkes, D.R., Quinn, J.B., Benoit, S.L., Shugarts, D.L., Griffin, A., Bakhtiari, M., Poticha, D., Eron, J.J., Fallon, M.A., & Rubin, M. 1996. Drug resistance and virologic response in NUCA 3001, a randomized trial of lamivudine (3TC) versus zidovudine (zidovudine) versus zidovudine plus 3TC in previously untreated patients. AIDS, 10, (9) 975-981 available from: PM:8853730
59. Larder, B.A. 1995. Viral resistance and the selection of antiretroviral combinations. J.Acquir.Immune.Defic.Syndr.Hum.Retrovirol., 10 Suppl 1, S28-S33 available from: PM:8595505
60. Phillips, A.N., Eron, J., Bartlett, J., Kuritzkes, D.R., Johnson, V.A., Gilbert, C., Johnson, J., Keller, A., & Hill, A.M. 1997. Correspondence between the effect of zidovudine plus lamivudine on plasma HIV level/CD4 lymphocyte count and the incidence of clinical disease in infected individuals. North American Lamivudine HIV Working Group. AIDS, 11, (2) 169-175 available from: PM:9030363
61. Wittkop, L., Gunthard, H.F., de, W.F., Dunn, D., Cozzi-Lepri, A., De, L.A., Kucherer, C., Obel, N., von, W., V, Masquelier, B., Stephan, C., Torti, C., Antinori, A., Garcia, F., Judd, A., Porter, K., Thiebaut, R., Castro, H., van Sighem, A.I., Colin, C., Kjaer, J., Lundgren, J.D., Paredes, R., Pozniak, A., Clotet, B., Phillips, A., Pillay,

D., & Chene, G. 2011. Effect of transmitted drug resistance on virological and immunological response to initial combination antiretroviral therapy for HIV (EuroCoord-CHAIN joint project): a European multicohort study. Lancet Infect.Dis., 11, (5) 363-371 available from: PM:21354861

62. Wittkop, L., Bitard, J., Lazaro, E., Neau, D., Bonnet, F., Mercie, P., Dupon, M., Hessamfar, M., Ventura, M., Malvy, D., Dabis, F., Pellegrin, J.L., Moreau, J.F., Thiebaut, R., & Pellegrin, I. 2013. Effect of cytomegalovirus-induced immune response, self antigen-induced immune response, and microbial translocation on chronic immune activation in successfully treated HIV type 1-infected patients: the ANRS CO3 Aquitaine Cohort. J.Infect.Dis., 207, (4) 622-627 available from: PM:23204178
63. Gallant, J.E., Staszewski, S., Pozniak, A.L., DeJesus, E., Suleiman, J.M., Miller, M.D., Coakley, D.F., Lu, B., Toole, J.J., & Cheng, A.K. 2004. Efficacy and safety of tenofovir DF vs stavudine in combination therapy in antiretroviral-naive patients: a 3-year randomized trial. JAMA, 292, (2) 191-201 available from: PM:15249568
64. Harrigan, P.R., Hogg, R.S., Dong, W.W., Yip, B., Wynhoven, B., Woodward, J., Brumme, C.J., Brumme, Z.L., Mo, T., Alexander, C.S., & Montaner, J.S. 2005. Predictors of HIV drug-resistance mutations in a large antiretroviral-naive cohort initiating triple antiretroviral therapy. J.Infect.Dis., 191, (3) 339-347 available from: PM:1563309
65. Ledergerber, B., Egger, M., Opravil, M., Telenti, A., Hirschel, B., Battegay, M., Vernazza, P., Sudre, P., Flepp, M., Furrer, H., Francioli, P., & Weber, R. 1999. Clinical progression and virological failure on highly active antiretroviral therapy in HIV-1 patients: a prospective cohort study. Swiss HIV Cohort Study. Lancet, 353, (9156) 863-868 available from: PM:10093977
66. Phillips, A.N., Staszewski, S., Weber, R., Kirk, O., Francioli, P., Miller, V., Vernazza, P., Lundgren, J.D., & Ledergerber, B. 2001. HIV viral load response to antiretroviral therapy according to the baseline CD4 cell count and viral load. JAMA, 286, (20) 2560-2567 available from: PM:11722270
67. Phillips, A.N., Dunn, D., Sabin, C., Pozniak, A., Matthias, R., Geretti, A.M., Clarke, J., Churchill, D., Williams, I., Hill, T., Green, H., Porter, K., Scullard, G., Johnson, M., Easterbrook, P., Gilson, R., Fisher, M., Loveday, C., Gazzard, B., & Pillay, D. 2005. Long term probability of detection of HIV-1 drug resistance after starting antiretroviral therapy in routine clinical practice. AIDS, 19, (5) 487-494 available from: PM:15764854
68. Staszewski, S., Miller, V., Sabin, C., Carlebach, A., Berger, A.M., Weidmann, E., Helm, E.B., Hill, A., & Phillips, A. 1999a. Virological response to protease inhibitor therapy in an HIV clinic cohort. AIDS, 13, (3) 367-373 available from: PM:10199227
69. Staszewski, S., Miller, V., Sabin, C., Schlecht, C., Gute, P., Stamm, S., Leder, T., Berger, A., Weidemann, E., Hill, A., & Phillips, A. 1999b. Determinants of sustainable CD4 lymphocyte count increases in response to antiretroviral therapy. AIDS, 13, (8) 951-956 available from: PM:10371176
70. Van Leth, L.F., Phanuphak, P., Ruxrungtham, K., Baraldi, E., Miller, S., Gazzard, B., Cahn, P., Lalloo, U.G., van der Westhuizen, I.P., Malan, D.R., Johnson, M.A., Santos, B.R., Mulcahy, F., Wood, R., Levi, G.C., Reboredo, G., Squires, K., Cassetti, I., Petit, D., Raffi, F., Katlama, C., Murphy, R.L., Horban, A., Dam, J.P., Hassink, E., van, L.R., Robinson, P., Wit, F.W., & Lange, J.M. 2004. Comparison of first-line antiretroviral therapy with regimens including nevirapine, efavirenz, or both drugs, plus stavudine and lamivudine: a randomised open-label trial, the 2NN Study. Lancet, 363, (9417) 1253-1263 available from: PM:15094269
71. Cambiano V, Bertagnolio S, Jordan M, Pillay D, Perriens J, Venter F, et al. Predicted levels of HIV drug resistance: potential impact of expanding diagnosis, retention, and eligibility criteria for antiretroviral therapy initiation. AIDS 2014, 28 (Suppl 1):S15–S23.
72. Ruggiero A, Cozzi-Lepri A, Beloukas A, Richman D, Khoo S, Phillips AN, et al. ERAS Study Group. Factors Associated With Persistence of Plasma HIV-1 RNA During Long-term Continuously Suppressive Firstline Antiretroviral Therapy. Open Forum Infectious Diseases 2018, 5 https://doi.org/10.1093/ofid/ofy032
73. Gross, R., Bilker, W.B., Friedman, H.M., & Strom, B.L. 2001. Effect of adherence to newly initiated antiretroviral therapy on plasma viral load. AIDS, 15, (16) 2109-2117 available from: PM:11684930
74. Ledergerber, B., Lundgren, J.D., Walker, A.S., Sabin, C., Justice, A., Reiss, P., Mussini, C., Wit, F., d'Arminio, M.A., Weber, R., Fusco, G., Staszewski, S., Law, M., Hogg, R., Lampe, F., Gill, M.J., Castelli, F.,

& Phillips, A.N. 2004. Predictors of trend in CD4-positive T-cell count and mortality among HIV-1-infected individuals with virological failure to all three antiretroviral-drug classes. Lancet, 364, (9428) 51-62 available from: PM:15234856.

75. A Mocroft, A N Phillips, J Gatell, B Ledergerber, M Fisher, N Clumeck,et al. Normalisation of CD4 counts in patients with HIV-1 infection and maximum virological suppression who are taking combination antiretroviral therapy: an observational cohort study Lancet 2007; 370: 407–13 Lancet 2007; 370: 407–13
76. Bishop J, DeShields S, Cunningham T, Troy SB. CD4 Count Recovery After Initiation of Antiretroviral Therapy in Patients Infected With Human Immunodeficiency Virus. Am JmedSci 2016;352(3):239–244
77. Geng E, Neilands T, Thiebaut R, Bosco Bwana M, Nash D, Moore R, et al. CD4 T cell recovery during suppression of HIV replication: an international comparison of the immunological efficacy of antiretroviral therapy in North America, Asia and Africa. International Journal of Epidemiology, 2015, 251–263 doi: 10.1093/ije/dyu271
78. O'Connor J, Smith CJ, Lampe FC, Hill T, Gompels M, Hay P, et al. Failure to achieve a CD4 cell count response on combination antiretroviral therapy despite consistent viral load suppression. AIDS 2014, 28:919–924
79. d'Arminio Monforte A, Cozzi Lepri A, Phillips AN, et al. Interruption of HAART in HIV clinical practice. Results from the ICONA study. JAIDS 2005; 38: 407-416
80. Li X, Margolick JB, Conover CS, et al. Interruption and discontinuation of HART in the MACS. JAIDS 2005; 38: 3:320-328.
81. Mocroft A, Youle M, Moore A, et al. Reasons for modification and discontinuation of antiretrovirals: results from a single treatment centre. AIDS 2001; 15 (2): 185-194.
82. Wit FWNM, Blanckenberg DH, Brinkman K, et al. Safety of long-term interruption of successful antiretroviral therapy: the ATHENA cohort study. AIDS 2005; 19: 345-348.
83. Sigaloff K, et a. Accumulation of HIV Drug Resistance Mutations in Patients Failing First-Line Antiretroviral Treatment in South Africa. AIDS Res Hum Retr 2012; 28:171-175.
84. Deeks SG, Grant RM, Wrin T, et al. Persistence of drug-resistant HIV-1 after a structured treatment interruption and its impact on treatment response. AIDS 2003; 17:361-370.
85. Devereux HL, Youle M, Johnson MA, et al Rapid decline in detectability of HIV-1 drug resistance mutations after stopping therapy. AIDS 1999; 13:F123-F127.
86. Devereux HL, Emery VC, Johnson MA, et al. Replicative fitness in vivo of HIV-1 variants with multiple drug resistance associated mutations. J Med Virol 2001:; 65:218-224.
87. Hance AJ, Lemiale V, Izopet J, et al. Changes in HIV-1 populations after treatment interruption in patients failing antiretroviral therapy. J Virol 2001; 75:6410-6417
88. Tarwater PM, Parish M, Gallant JE. Prolonged treatment interruption after immunologic response to HAART. Clin Infect Dis 2003; 37:1541-1548.
89. Walter H, Low P, Harrer T, et al. No evidence for persistence of multidrug resistant viral strains after a 7-month treatment interruption in an HIV-1 infected individual. JAIDS 2002; 31:137-146
90. Birk M, Svedhem V, Sonnerborg A. Kinetics of HIV-1 RNA and resistance-associated mutations after cessation of antiretroviral combination therapy. AIDS 2001; 15:1359-1368
91. Castro H, Pillay D, Cane P, Asboe A, Cambiano V, Phillips AN, Dunn DT. Persistence of Transmitted HIV-1 Drug Resistance Mutations. JID 2013; DOI: 10.1093/infdis/jit345
92. Smith DM, Wong JK, Hightower GK, et al. HIV drug resistance acquired through superinfection. AIDS 2005; 19: 1251–56.
93. Corvasce et al. Evidence of differential selection of HIV-1 variants carrying drug-resistant mutations in seroconverters. Antiviral Therapy 2006; 11:329 -334.
94. Turner et al. Diminished Representation of HIV-1 Variants Containing Select Drug Resistance–Conferring mutations in Primary HIV-1 Infection. JAIDS 2004; 37: 1627-1631)
95. Phillips AN, Cambiano V, Nakagawa F, Brown AE, Lampe F, et al. (2013) Increased HIV Incidence in Men Who Have Sex with Men Despite High Levels of ART-Induced Viral Suppression: Analysis of an Extensively Documented Epidemic. PLoS ONE 8(2): e55312. doi:10.1371/journal.pone.0055312

96. Phillips A, CASCADE Collaboration. Short-term risk of AIDS according to current CD4 cell count and viral load in antiretroviral drug-naïve individuals and those treated in the monotherapy era. AIDS, 2004. 18(1):51-8.
97. Johnson LF, et al. Sexual behaviour patterns in South Africa and their association with the spread of HIV: Insights from a mathematical model. Demographic Research 2009; 21:289-340
98. Gregson S et al. Methods to reduce social desirability bias in sex surveys in low-development settings - Experience in Zimbabwe. Sexually Transmitted Diseases 2002; 29: 568-575.
99. Gregson S, Gonese E, Hallett TB, et al. HIV decline in Zimbabwe due to reductions in risky sex? Evidence from a comprehensive epidemiological review. Int J Epid 2010;39:1311–1323.
100. Halperin DT, Mugurungi O, Hallett TB, Muchini B, Campbell B, et al. (2011) A Surprising Prevention Success: Why Did the HIV Epidemic Decline in Zimbabwe? PLoS Med 8(2): e1000414. doi:10.1371/journal.pmed.1000414
101. Fonner VA, Denison J, Kennedy CE, O'Reilly K, Sweat M. Voluntary counseling and testing (VCT) for changing HIVrelated risk behavior in developing countries. Cochrane Database of Systematic Reviews 2012, Issue 9. Art. No.: CD001224. DOI:10.1002/14651858.CD001224.pub4.
102. Afonso JM, Bello G, Guimarães ML, Sojka M, Morgado MG. HIV-1 genetic diversity and transmitted drug resistance mutations among patients from the North, Central and South regions of Angola. PLoS ONE. 2012;7(8):e42996.
103. Rowley CF, MacLeod IJ, Maruapula D, Lekoko B, Gaseitsiwe S, Mine M et al. Sharp increase in rates of HIV transmitted drug resistance at antenatal clinics in Botswana demonstrates the need for routine surveillance. J Antimicrob Chemother. 2016;71(5):1361–6.
104. National Institute for Communicable Diseases, Division of the National Health Laboratory Service. Prospective sentinel surveillance of human immunodeficiency virus related drug resistance. Communicable Disease Communiqué. 2016 March; 15:10-11. http://nicd.ac.za/assets/files/NICD%20Communicable%20Diseases%20Communique_Mar2016_final.pdf
105. Pantazis N, Touloumi G. Bivariate modelling of longitudinal measurements of two human immunodeficiency type 1 disease progression markers in the presence of informative drop-outs. JRSS C 2005; 54: 405-423.
106. Sabin CA, Devereux H, Phillips AN, et al. Course of viral load throughout HIV-1 infection. JAIDS 2000; 23:172-177.
107. Hubert J-B, Burgard M, Dussaix E, et al. Natural history of serum HIV-1 RNA levels in 330 patients with known date of infection. AIDS 2000; 14:123-131.
108. O'Brien TR, Rosenberg PS, Yellin F, et al. Longitudinal HIV-1 RNA levels in a cohort of homosexual men. JAIDS 1998; 18:155-161.
109. Henrard DR, Phillips JF, Muenz LR et al. Natural history of HIV-1 cell-free viraemia. JAMA 1995; 274: 554-558.
110. Lyles RH, Munoz A, Yamashita TE, et al. Natural history of human immunodeficiency virus type 1 viraemia after seroconversion and proximal to AIDS in a large cohort of homosexual men. J Infect Dis 2000; 181 (3): 872-880.
111. Touloumi G, Pantazis N, Babiker AG, et al. Differences in HIV RNA levels before the initiation of antiretroviral therapy among 1864 individuals with known HIV-1 seroconversion dates. AIDS 2004; 18 (12): 1697-1705
112. Koot M, Keet IPM, Vos AHV, et al. Prognostic value of human HIV-1 biological phenotype for the rate of CD4+ cell depletion and progression to AIDS. Ann Intern Med 1993; 118: 681-688.
113. Mellors JW, Munoz A, Giorgi JV, et al. Plasma viral load and CD4(+) lymphocytes as prognostic markers of HIV-1 infection. Ann Intern Med 1997; 126 (12): 946-954.
114. Rosen S, Fox MP. Retention in HIV Care between Testing and Treatment in Sub-Saharan Africa: A Systematic Review. PLOS Medicine 2011; 8 Article Number: e1001056
115. Jain V, Sucupira MC, Bacchetti P, Hartogensis W, Diaz RS, Kallas EG, et al. Differential Persistence of Transmitted HIV-1 Drug Resistance Mutation Classes. J Infect Dis 2011; 203(8):1174-1181.

116. Yang W-L, Kouyos RD, Böni J, Yerly S, Klimkait T, Aubert V, et al. Persistence of Transmitted HIV-1 Drug Resistance Mutations Associated with Fitness Costs and Viral Genetic Backgrounds. PLoS Pathog 2015 11(3): e1004722. doi:10.1371/journal.ppat.1004722
117. McMahon JH, Spelman T, Ford N, Greig J, Mesic A, Ssonko C et al. Risk factors for unstructured treatment interruptions and association with survival in low to middle income countries. AIDS Res and Therapy 2016; 13 Article Number: 25
118. Agbaji OO, Abah IO, Falang KD, Ebonyi AO, Musa J, Ugoagwu P, et al. Treatment Discontinuation in Adult HIV-Infected Patients on First-Line Antiretroviral Therapy in Nigeria. Curr HIV Research 2015; 13: 184-192 DOI: 10.2174/1570162X1303150506181945
119. Tenores Study Group. Global epidemiology of drug resistance after failure of WHO recommended fi rst-line regimens for adult HIV-1 infection: a multicentre retrospective cohort study. Lancet Infect Dis 2016 http://dx.doi.org/10.1016/S1473-3099(15)00536-8
120. Clotet B, Feinberg J, van Lunzen J, Khuong-Josses MA, Antinori A, Dumitru I, et al. Once-daily dolutegravir versus darunavir plus ritonavir in antiretroviral-naive adults with HIV-1 infection (FLAMINGO): 48 week results from the randomised open-label phase 3b study. Lancet. 2014; 383(9936):2222–31. doi: 10.1016/S0140-6736(14)60084-2 PMID: 24698485
121. Wainberg MA, Han YS. Will drug resistance against dolutegravir in initial therapy ever occur? Front Pharmacol. 2015; 6:90. doi: 10.3389/fphar.2015.00090 PMID: 25972810
122. Wijting, Ingeborg; Rokx, Casper; Boucher, Charles; et al. Dolutegravir as maintenance monotherapy for HIV (DOMONO): a phase 2, randomised non-inferiority trial. LANCET HIV Volume: 4 Issue: 12 Pages: E547-E554 Published: DEC 2017
123. van Lunzen J, Maggiolo F, Arribas JR, Rakhmanova A, Yeni P, Young B, et al. Once daily dolutegravir (S/GSK1349572) in combination therapy in antiretroviral-naive adults with HIV: planned interim 48 week results from SPRING-1, a dose-ranging, randomised, phase 2b trial. Lancet Infect Dis 2012; 12: 111–18
124. Blanco, Jose L.; Marcelin, Anne-Genevieve; Katlama, Christine; et al. Dolutegravir resistance mutations: lessons from monotherapy studies CURRENT OPINION IN INFECTIOUS DISEASES Volume: 31 Issue: 3 Pages: 237-245 Published: JUN 2018
125. Katlama C, Soulie C, Caby F, Denis A, Blanc C, Schneider L, et al. Dolutegravir as monotherapy in HIV-1-infected individuals with suppressed HIV viraemia. JOURNAL OF ANTIMICROBIAL CHEMOTHERAPY 2016; 71: 2646-2650 DOI: 10.1093/jac/dkw186.
126. Lanzafame M, Gibellini D, Lattuada E, Signoretto C, Mazzi R, Concia E, et al. Dolutegravir Monotherapy in HIV-Infected Naive Patients With < 100,000 Copies/mL HIV RNA Load. JAIDS 2016; 72:E12-E14
127. Gubavu C, Prazuck T, Niang M, Buret J, Mille C, Guinard J, Avettand-Fenoel V, Hocqueloux L et al. Dolutegravir-based monotherapy or dual therapy maintains a high proportion of viral suppression even in highly experienced HIV-1-infected patients. J Antimicr Chem 2016; 71: 1046-1050. DOI: 10.1093/jac/dkv430
128. Borghetti A, Baldin G, Ciccullo A, Gagliardini R, Davino A, Mondi A, et al. Virological control and metabolic improvement in HIV-infected, virologically suppressed patients switching to lamivudine/dolutegravir dual therapy. J Antimicrob Chem 2016; 71: 2359-2361. DOI: 10.1093/jac/dkw147
129. Nicolè S, Lanzafame M, Lattuada E, Mazzi R, Rigo F, Cucchetto G, et al. Dolutegravir montherapy in HIV-infected naïve patients with <100,000 copies/ML HIV RNA load, an update of a little cohort in Verona. Infect Dis Trop Med 2016; 2 (2): e295.
130. Marcellin A, Grude G, Charpentier C, Bellecave P, Rodallec A, Pallier C, et al. French survey, resistance to integrs inhib shows differences of resistance selection in virological failure in care. Abst 332. HIV Glasgow Octover 2016.
131. Rutherford GW, Horvath H. Dolutegravir Plus Two Nucleoside Reverse Transcriptase Inhibitors versus efavirenz Plus Two Nucleoside Reverse Transcriptase Inhibitors As Initial Antiretroviral Therapy for People with HIV: A Systematic Review. PLoS ONE 2016 11(10): e0162775. doi:10.1371/journal.pone.0162775

132. Sax PE, DeJesus E, Crofoot G, Ward D, Benson P, Dretler R. Bictegravir versus dolutegravir, each with emtricitabine and TDF alafenamide, for initial treatment of HIV-1 infection: a randomised, double-blind, phase 2 trial. Lancet HIV 2017; 4: e154–60
133. Stellbrink H, Reynes J, Lazzarin A, Voronin E, Pulido F, Felizarta F, et al. Dolutegravir in antiretroviral-naive adults with HIV-1: 96-week results from a randomized dose-ranging study. AIDS 2013; 27:1771–1778
134. Patel DA, Snedecor SJ, Tang WY, Sudharshan L, Lim JW, et al. 48-Week Efficacy and Safety of Dolutegravir Relative to Commonly Used Third Agents in Treatment-Naive HIV-1–Infected Patients: A Systematic Review and Network Meta-Analysis. PLoS ONE 2014 9(9): e105653. doi:10.1371/journal.pone.0105653.
135. de Boer M, van den Berk GEL, van Holten N, Oryszcyn JE, Dorama W, ait Mohab D, et al. Intolerance of dolutegravir-containing combination antiretroviral therapy regimens in real-life clinical practice. AIDS 2016, 30:2831–2834.
136. Hoffmann C, Welz T, Sabranski M, Kolb M, Wolf E, Stellbrink1 H-J et al. Higher rates of neuropsychiatric adverse events leading to dolutegravir discontinuation in women and older patients. HIV Medicine 2017; 18, 56—63.
137. Menard A, Montagnac C, Solas C, Meddeb L, Dhiver D, Tomeia C, et al. Neuropsychiatric adverse effects on dolutegravir: an emerging concern in Europe AIDS 2017, Vol 31 No 8
138. Taha H, Das A, Das S. Clinical effectiveness of dolutegravir in the treatment of HIV/AIDS. Infect Drug Resist. 2015;8:339–52. doi: 10.2147/IDR.S68396. pmid:26491363
139. Llibre JM, Pulido F, Garcia F, Garcia Deltoro M, Blanco JL, Delgado R. Genetic barrier to resistance for dolutegravir. AIDS Rev. 2015; 17(1):56–64. PMID: 25472016 7.
140. Veloso Meireles M, Ana Roberta P. Pascom ARP , Duarte EC, McFarland W. Comparative effectiveness of first-line antiretroviral therapy: results from a large real-world cohort after the implementation of dolutegravir. AIDS 2019, 33:1663–1668
141. Cournil A, Kouanfack C, Eymard-Duvernay S, et al. Dolutegravir versus an efavirenz 400 mg-based regimen for the initial treatment of HIV-infected patients in Cameroon: 48-week efficacy results of the NAMSAL ANRS 12313 trial HIV Glasgow 2018; Glasgow, Scotland; Oct 28–31, 2018. Abstr O342.
142. ADVANCE study IAS meeting Mexico City July 2019
143. Msyamboza KP, Ngwira B, Dzowela T, Mvula C, Kathyola D, et al. (2011) The Burden of Selected Chronic Non-Communicable Diseases and Their Risk Factors in Malawi: Nationwide STEPS Survey. PLoS ONE 6(5): e20316. doi:10.1371/journal.pone.0020316
144. Thorogood M, Connor M, Tollman S, Lewando Hundt G, Fowkes G, Marsh J. A cross-sectional study of vascular risk factors in a rural South African population: data from the Southern African Stroke Prevention Initiative (SASPI). BMC Public Health 2007, 7:326 doi:10.1186/1471-2458-7-326
145. Berrington de Gonzalez A, Hartge P, Cerhan JR, Flint AJ, Hannan L, MacInnis RJ. Body-Mass Index and Mortality among 1.46 Million White Adults. N Engl J Med. 2010 December 2; 363(23): 2211–2219. doi:10.1056/NEJMoa1000367.
146. Flegal KM, Kit BK, Orpana H, Graubard BI. Association of All-Cause Mortality With Overweight and Obesity Using Standard Body Mass Index Categories:A Systematic Review and Meta-analysis. JAMA. 2013 January 2; 309(1): 71–82. doi:10.1001/jama.2012.113905.
147. Achhra AC, Sabin CA, Ryom L, Hatleberg C, d'Aminio Monforte, de Wit S, et al. Body Mass Index and the Risk of Serious Non-AIDS Events and All-Cause Mortality in Treated HIV-Positive individuals: D:A:D Cohort Analysis. J Acquir Immune Defic Syndr _ Volume 78, Number 5, August 15, 2018.
148. Kivimäki M, Kuosma , Ferrie JF, Luukkonen R, Nyberg ST, Alfredsson L, et al. Overweight, obesity, and risk of cardiometabolic multimorbidity: pooled analysis of individual-level data for 120 813 adults from 16 cohort studies from the USA and Europe. Lancet Public Health 2017; 2: e277–85.
149. Meta-analysis of association between baseline NNRTI resistance and virologic failure prepared for WHO guidelines meeting June 2019.
150. Cresswell J, Campbell OMR, De Silva MJ, Filippi V. Effect of maternal obesity on neonatal death in sub-Saharan Africa: multivariable analysis of 27 national datasets. Lancet 2012; 380: 1325–30

151. Salomon JA, Vos T, Hogan DR, et al. Common values in assessing health outcomes from disease and injury: disability weights measurement study for the Global Burden of Disease Study 2010. Lancet 2012; 380: 2129–43.
152. Global Fund Pooled Procurement Price list July 2018. https://www.theglobalfund.org/media/5813/ppm_arvreferencepricing_table_en.pdf
153. Eaton J et al. Health benefits, costs, and cost-effectiveness of earlier eligibility for adult antiretroviral therapy and expanded treatment coverage: a combined analysis of 12 mathematical models. Lancet Global Health 2014: E23-E34
154. Hyle, E. P., Jani, I. V, Lehe, J., Su, A. E., Wood, R., Quevedo, J., … Walensky, R. P. (2014). The Clinical and Economic Impact of Point-of-Care CD4 Testing in Mozambique and Other Resource-Limited Settings: A Cost-Effectiveness Analysis. PLoS Med, 11(9), e1001725. doi:10.1371/journal.pmed.1001725.
155. Keebler D, Revill P, et al. How Should HIV Programmes Monitor Adults on ART? A Combined Analysis of Three Mathematical Models. Lancet Global Health 2014. E35-E43.
156. Global Fund Releases. http://www.theglobalfund.org/en/mediacenter/newsreleases/2015-06-10_New_Approach_on_HIV_Viral_Load_Testing/ http://www.theglobalfund.org/en/procurement/viral-load-early-infant-diagnostics/)
157. CHAI 2018 Market Report. https://clintonhealthaccess.org/content/uploads/2018/09/2018-HIV-Market-Report_FINAL.pdf
158. Siapka M, Remme M, Dayo Obure C, Maier C, Dehne KL, Vassall A. Is there scope for cost savings and efficiency gains in HIV services? A systematic review of the evidence from low- and middle-income countries. Bull World Health Organ 2014;92:499–511AD doi:http://dx.doi.org/10.2471/BLT.13.127639;
159. Tagar E, Sundaram M, Condliffe K, Matatiyo B, Chimbwandira F, et al. Multi-Country Analysis of Treatment Costs for HIV/AIDS (MATCH): Facility-Level ART Unit Cost Analysis in Ethiopia, Malawi, Rwanda, South Africa and Zambia. PLoS ONE 2014; 9(11): e108304. doi:10.1371/journal.pone.0108304;
160. Menzies NA, Berruti AA, Blandford JM (2012) The Determinants of HIV Treatment Costs in Resource Limited Settings. PLoS ONE 7(11): e48726. doi:10.1371/journal.pone.0048726
